# CyTOF^®^ for the Masses

**DOI:** 10.3389/fimmu.2022.815828

**Published:** 2022-04-14

**Authors:** Akshay Iyer, Anouk A. J. Hamers, Asha B. Pillai

**Affiliations:** ^1^ Department of Pediatrics, University of Miami Miller School of Medicine, Miami, FL, United States; ^2^ Department of Microbiology and Immunology, University of Miami Miller School of Medicine, Miami, FL, United States; ^3^ Sylvester Comprehensive Cancer Center, University of Miami Miller School of Medicine, Miami, FL, United States; ^4^ Sheila and David Fuente Program in Cancer Biology, University of Miami Miller School of Medicine, Miami, FL, United States

**Keywords:** mass cytometry, workflow, experimental design, protocol, data analysis, panel design, barcoding, CyTOF

## Abstract

Mass cytometry has revolutionized immunophenotyping, particularly in exploratory settings where simultaneous breadth and depth of characterization of immune populations is needed with limited samples such as in preclinical and clinical tumor immunotherapy. Mass cytometry is also a powerful tool for single-cell immunological assays, especially for complex and simultaneous characterization of diverse intratumoral immune subsets or immunotherapeutic cell populations. Through the elimination of spectral overlap seen in optical flow cytometry by replacement of fluorescent labels with metal isotopes, mass cytometry allows, on average, robust analysis of 60 individual parameters simultaneously. This is, however, associated with significantly increased complexity in the design, execution, and interpretation of mass cytometry experiments. To address the key pitfalls associated with the fragmentation, complexity, and analysis of data in mass cytometry for immunologists who are novices to these techniques, we have developed a comprehensive resource guide. Included in this review are experiment and panel design, antibody conjugations, sample staining, sample acquisition, and data pre-processing and analysis. Where feasible multiple resources for the same process are compared, allowing researchers experienced in flow cytometry but with minimal mass cytometry expertise to develop a data-driven and streamlined project workflow. It is our hope that this manuscript will prove a useful resource for both beginning and advanced users of mass cytometry.

## Introduction

Mass cytometry, also termed cytometry by Time-Of-Flight (CyTOF^®^), is a powerful tool for high-dimensional and high-throughput single-cell assays. First introduced in 2009 by Bandura et al. (1), mass cytometry has become an important tool in the analysis of immune cell function/activation due to its high-parameter capabilities. Since 2015, the application of mass cytometry for immunophenotyping in hematopoietic stem cell transplantation (HSCT) (2–6), tumor microenvironment (TME) (7–13) and cancer immunotherapy (6, 9, 14–17) has significantly expanded.

Until recently, fluorescent-based (conventional) flow cytometry was the method of choice for phenotypic and functional analysis of single cells. Standard flow cytometry technologies using 4- or 5-laser data acquisition instruments allow analysis of up to 30 parameters simultaneously. The newer fluorescent-based flow cytometry machines (spectral flow cytometers) measure the total fluorescence in 1 sample and then use an unmixing technology to mathematically separate the specific fluorophore signals (18). These data acquisition machines can process up to 50 parameters simultaneously; however, practical application typically allows a maximum of 40 parameters (19). Due to the broader emission spectra of fluorescent probes following laser excitation, overlapping emission spectra remains a significant issue in flow cytometry. Mass cytometry replaces fluorescent labels with non-biologically available metal isotopes with concise mass spectrometry parameters, thereby overcoming the pitfalls associated with overlapping emission spectra and increasing the number of simultaneously analyzable parameters further (20, 21). In mass cytometry, cells are incubated with a mixture of probes/antibodies tagged with a unique non-radioactive heavy metal isotope. Single-cell suspensions are nebulized such that each droplet contains a single cell. Individual cells subsequently pass through argon (Ar) plasma, which atomizes and ionizes the sample. This converts each cell into a cloud containing ions of the elements present in or on that cell. A high-pass optic (quadrupole) removes the low-mass (mainly biologic) ions from each cloud (ions with mass below 75 Da), resulting in a cloud containing only those ions corresponding to the isotope-conjugated probes. In the Time of Flight (TOF) chamber, the ions are separated by mass-to-charge ratio. Upon encountering the detector, these ion counts are amplified and converted into electrical signals. Theoretically, 120 parameters can be studied simultaneously. However, realistically the availability of isotopes with sufficient purity as well as antibody conjugation chemistries limit applications to ~60 parameters per mass cytometry panel. A single-cell technology generating even more dimensions is single-cell RNA sequencing (scRNAseq), which gives a quantitative measure of gene expression levels per cell. scRNAseq is a powerful genomic tool for dissecting cell populations. However, scRNAseq can only be run on a small number of single cells (limited mainly by increased costs), whereas mass cytometry experiments can acquire data on several times that number (in the range of 10^6^-10^7^ cells), facilitating the characterization of rare cell populations. Additionally, mass cytometry can add critical functional information through protein analyses. When the complementary techniques of scRNAseq and mass cytometry are combined, one can rigorously phenotypically and functionally characterize diverse cell populations within a single sample. Mass cytometry can also be used to confirm data derived from scRNAseq. Considering the complexity of the TME, such a multimodal approach yields powerful data applicable to both tumor-intrinsic and tumor-extrinsic effects of immunotherapies in the TME, as well as the correlation of peripheral immune signatures with treatment response or failure or identification of new targets (22).

**Figure 1:**
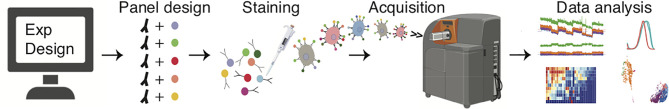
Typical workflow used in mass cytometry experiments. An experiment starts with careful design of an antibody/probe panel. This is followed by sample processing, staining and acquisition, and finally data analysis.

The purpose of this article is to detail considerations critical to designing and performing a mass cytometry experiment for immunologists and cancer biologists with limited expertise (**Figure 1**). Our target audience includes not only bench scientists and clinicians with knowledge of basic flow cytometry, but also computational scientists and immunotherapy-focused individuals working with mass cytometry datasets. For more detailed reference literature on conventional flow cytometry, we refer the reader to selected reviews, guidelines, and protocols (18, 23–27).

## Experimental Design

### Study Endpoints and Sample Sources

Paramount to carefully planning mass cytometry experiment design is consideration of the study goal (e.g. identifying multiple new populations in a sample, characterizing an unknown cell population, proportional comparison of multiple well-characterized cell populations, novel biomarker discovery, and analysis of protein expression, cell cycle or phosphorylation state, and pharmacokinetics/pharmacodynamics). The large number of parameters simultaneously analyzable by mass cytometry facilitates assessing all of these endpoints leveraging a single antibody panel.

In addition, it is important to plan ahead for the sample sources to be used. Each sample source and tissue type has corresponding optimal pre-analysis sample processing and storage considerations; the researcher is referred to existing interactive resources noted elsewhere in this review to raise project-specific questions for clarification.

Many experimental factors can affect mass cytometry data, including cell isolation, staining protocol, fixation, and donor-specific biological variation. All of these are described in more detail by *Olsen *et al. (28) Accounting for these factors, isolation and staining protocols may need several optimization rounds and unique quality controls. An important quality control (QC) approach for reproducibility and staining consistency is an internal control per sample tube, which can be achieved by sample barcoding (see *Barcoding*). Another QC element when studying cytokines or transcription factor activation is stimulated versus unstimulated conditions. The sample distribution itself can contribute important QC components, since all cell types are not positive for all markers (i.e. internal positive and negative controls).

### Isotope- Antibody Pairing

**Figure 2:**
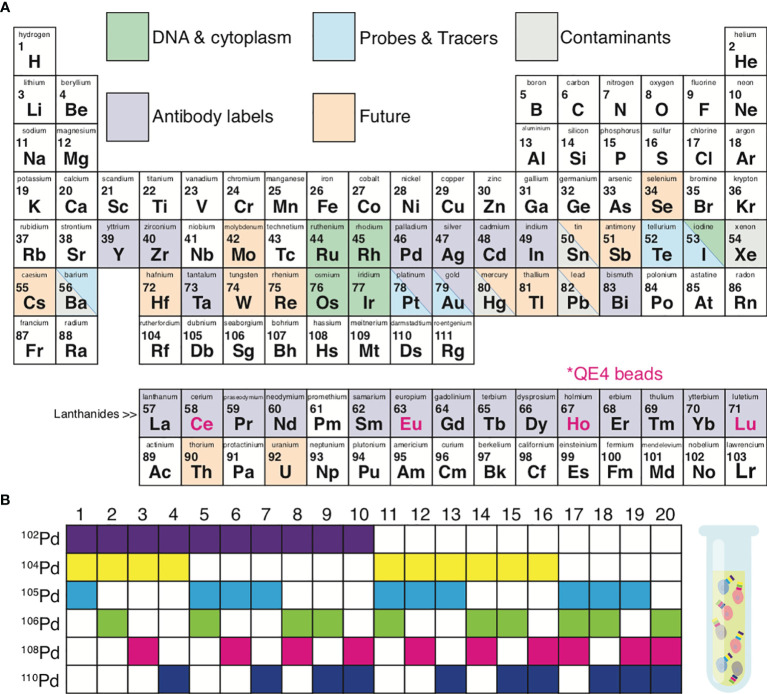
Metal isotopes utilized in mass cytometry. **(A)** Periodic table summarizing the elements currently available for mass cytometry experiments. *Grey*, possible contaminating elements; *green*, elements used to indicate DNA content or cell size; *purple*, elements available for antibody conjugations; *blue*, elements available as probes and tracers; *orange*, elements not yet explored for mass cytometry but of potential future interest. *Pink font*; isotopes included in QE4 calibration beads. **(B)** Certain isotopes work well for the use in sample 1 barcoding. Shown is an example of palladium barcoding using 6 isotopes in unique combinations of 3. This strategy generates 20 separate barcodes, allowing 20 individual samples to be combined into 1 single tube.

Proper pairing of antibodies with metal isotopes (see *Panel Design*) is critical. When opting for standard pre-conjugated antibodies, the main limitation is the extent of the vendor portfolio. Alternatively, lanthanides can be conjugated *de novo* to purified antibodies using a Maxpar^®^ X8 antibody labelling kit (Fluidigm, San Francisco, CA) (29–31). With lanthanides, a panel can contain up to 37 cellular markers/antibodies (**Figure 2A**). One limitation of this approach is that proteins such as bovine serum albumin (BSA) (often used in the buffers of purified antibodies) can bind lanthanides, resulting in failed antibody conjugation due to adsorption of the lanthanide.

In addition to lanthanides, the distinct isotopes of cadmium (31), palladium (32, 33), indium (32), platinum (34), and bismuth (32, 35) can be utilized, to a total of 60 distinct isotopes (**Figure 2A**). The Maxpar^®^ MCP9 antibody labelling kit (Fluidigm, San Francisco, CA) is specifically designed to conjugate cadmium (Cd) isotopes (31). Most commercial Qdots used for conventional flow cytometry contain isotopes of cadmium (^106-116^Cd) with either selenium (^72-82^Se) or tellurium (^120-130^Te), which are readily available as antibody conjugates (36, 37). More recent options for antibody labels include streptavidin-coated gold or silver nanoparticles (38, 39) and tantalum oxide nanoparticles (40).

### Barcoding

Sample throughput can be enhanced, costs reduced, and data quality improved by utilizing sample barcoding (33, 41). Each experimental sample (e.g. across individuals or treatment groups) can be tagged with a unique isotope of a particular element (e.g., after which all samples are combined into 1 tube. **Figure 2B** demonstrates a 6-choose-3 barcoding example. Six Palladium (Pd) isotopes can be used to generate 20 different barcodes, where each barcode is created from a combination of any 3 Pd isotopes. Barcoding minimizes the possibility for inter-sample staining variability, reduces cell-cell doublets, and minimizes the propensity for inter-sample cross-contamination seen in serial runs across individual samples (33, 42–44). Sample barcoding minimizes inter-sample staining variability by avoiding sample-to-sample pipetting errors and inconsistent incubation times. In a 6-choose-3 barcoding scheme, a cell-cell doublet will yield an illegal barcode (I.e. a combination of 2 existing barcodes from the scheme) with a positivity for at least 4 out of 6 isotopes, which cannot belong to a single cell event (33). Doublets between cells within a sample cannot be detected and removed by barcoding alone. Moreover, the use of all possible Pd combinations (any combination of 6 isotopes generating 64 barcodes) can result in miscoding if one or more reagents fail, making it impossible to exclude cell-cell doublets. Sample barcoding can also be utilized to add an internal control into each tube prior to staining. This internal control generates the same results/clustering/cell proportions from one tube to the next and is therefore strongly recommended for optimal data quality and reproducibility. Internal control cells can be cryopreserved and rethawed pooled wild-type mouse splenocytes or PBMC from the same healthy donor or Veri-cells^®^ (Biolegend, San Diego, CA) (45, 46).

There are 3 main options in sample barcoding:

1. The Cell-ID^®^ 20-Plex palladium (Pd) barcoding kit (Fluidigm, San Francisco, CA) uses 6 distinct Pd isotopes to combine up to 20 samples per tube (41, 47, 48). This is an intracellular method; one limitation is that samples need to be fixed prior to panel staining, so epitopes may be crosslinked in such a way that the corresponding antibody no longer recognizes the intended epitope. Therefore, protocol troubleshooting and optimization is needed prior to applying this approach.
2. Antibody-based live cell barcoding is more flexible as it does not require fixation prior to panel staining. Unique Pd, cadmium (Cd), and/or platinum (Pt) isotopes are conjugated to antibodies directed against ubiquitous epitopes such as CD45 (hematopoietic lineages), β2 microglobulin (class I MHC and CD1 isomers), or CD298 (integral membrane cationic ATPase-associated proteins), and samples are stained with different combinations of these antibodies (42, 44, 49, 50). Pd, Cd, or Pt are ideal for live cell barcoding primarily because these isotopes are outside the CyTOF^®^ optimum mass range of 153 to 176 and therefore tend to be less “bright” (51). Pd and Cd isotopes are well below and Pt is well above the 139-176 mass range of lanthanides and therefore do not influence lanthanide-based antibody detection. Pd live cell barcoding is more labor intensive than Cd, since there is no available kit for Pd conjugations. Recently, *Muftuoglu *et al. (52) showed that Cd-CD45 barcodes elicit higher signal intensities than Pd-CD45 barcodes, most likely attributable to superior signal resolution because MCP9 polymers used to conjugate Cd chelate a higher number of isotopes as compared to mDOTA (used to conjugate Pd). (This group also showed that it is possible to conjugate Pd isotopes to CD45 antibodies using MCP9, and that this results in an equal signal intensity as for the Cd-CD45 conjugates.)
3. Monoisotopic cisplatin-based live cell barcoding is the simplest and fastest method available. Cisplatin is used to directly label cells, without the need for antibody conjugations (53, 54). Cisplatin, a chemotherapeutic agent, contains platinum and is available from Fluidigm as any of the following isotopes: ^194^Pt, ^195^Pt, ^196^Pt, and ^198^Pt.

Thiol-reactive tellurium (TeMal) (55) or osmium and ruthenium tetroxide (56) can be added to any of the 3 barcoding strategies in order to further increase multiplexing capabilities.

### Cell Numbers and Viability

Mass cytometry sample staining and acquisition induces a high rate of cell loss. Therefore, starting with 800,000 - 1 million cells per sample is advisable. Typically, only 50-70% of the sample can be recovered in the data; the remainder is loss due to aggregation on the walls of the spray chamber and injector (28, 57). Of note, these numbers are based on the CyTOF Helios^®^ instrument (Fluidigm, San Francisco, CA). With the CyTOF2^®^ instrument (Fluidigm, San Francisco, CA), cell recovery is even lower (30-40%) (20). Of note, this cell loss inside the machine is stochastic in nature and therefore does not appear to introduce sampling bias (51).

Optimal starting cell numbers are highly dependent on the study and the planned sample staining protocol. Studies involving rare cell populations or transcription factors require a larger starting sample size for adequate rigor as compared to studies investigating prevalent subsets. During sample processing and staining, an additional cell loss of 20-30% must be considered.

It is important to minimize inter-sample variability in analyzed cell number and viability in order to insure reproducible staining approaches across experiments. When processing samples, tissue digestions, freeze/thaw cycles, and incomplete fixation prior to permeabilization can introduce sampling bias by differentially affecting specific cell populations. Dead cells may compromise flow and mass cytometric data by non-specifically trapping antibodies (58). In addition, dead cells tend to release DNA, which adheres to cells, causes cell aggregation, and increases cell doublets. Tissue digestions can also cause an overall low cell viability; achieving a high cell viability (> 80%) is important in ensuring high-quality data (59, 60). To address low cell viability, dead cell removal kits are available [e.g. Miltenyi Biotec Inc (Auburn, CA) and STEMCELL Technologies Inc (Cambridge, MA)] for application prior to sample staining to assist in data QC.

A convenient solution for low cell yields prior to sample staining is live cell barcoding. Not only can multiple low-yield samples be combined in a single tube, but a spiked-in internal control can also be added, increasing the total analysis cell number and distributing cell loss in downstream steps across both study samples and controls and thereby preserving a greater fraction of the study sample (7, 45). For example, *Winkels *et al. (61) combined barcoded mouse splenocytes with mouse aorta samples, preserving a greater fraction of the murine aorta samples.

Where low cell yields and/or poor viability persist, the cellular composition of these tissues can instead be studied *in situ* using a histologic approach. There are 2 platforms available for this purpose. In imaging mass cytometry [Hyperion^®^ (Fluidigm)], a laser ablates histological sections stained with metal-labelled antibodies (62, 63). A more novel metal-based histology platform with increased speed, sensitivity, and image resolution is multiplexed ion beam imaging [MIBI (Ionpath)], which collects data through secondary ions released from the histological slide by primary ion beams (64–66).

## Panel Design & Antibody Conjugation

As in conventional flow cytometry, panel design is key to mass cytometry experiment success (36, 57, 67). The initial marker selection relies heavily on the scientist’s combined biological knowledge and familiarity with statistical testing methods, varying depending on the sample type, cell type, and overall experimental objectives. Relevant biological knowledge includes that from literature and from data generated from prior RNA sequencing or conventional flow cytometry. Marker screen kits are available (68, 69). Beyond isotope-conjugated antibodies, other probes which can be included in mass cytometry panels include tetramers (70), carbohydrate-binding molecules (71), tellurium-based oxygen sensors (72), inorganic nanoparticles (73), RNA probes (74, 75), and modified nucleotides (75, 76) (**Figure 2A**). With the exception of oxygen (72), small molecules or proteins were not detectable by mass cytometry until the Nitz group developed a Tellurium-containing analog of phenylalanine, making it possible to monitor protein synthesis (77). Poreba et al. have since developed multiple protease-selective lanthanide-labelled probes for mass cytometry (78).

Since cells are atomized and ionized inside a mass cytometer, the resulting data lacks the Side Scatter (SSC) and Forward Scatter (FSC) parameters used for cell doublet and debris discrimination in conventional flow cytometry. Therefore, mass cytometry relies on the use of a DNA intercalator (see *Sample Staining*) (7). As an alternative to the FSC parameter, Osmium Tetroxide (OsO_4_) has been suggested as a useful tool to reconstruct cell size in mass cytometry data (79). OsO4 is a nonpolar compound that penetrates charged membranes and can be detected directly by the mass cytometer. *Good* et al. have also adapted carboxy-fluorescein succinimidyl ester (CFSE)-based protocols for tracking cell proliferation in mass cytometry using a metal-conjugated CFSE cross-reactive anti-fluorescein isothiocyanate (anti-FITC) antibody (80).

The next step in panel design is pairing an antibody or probe with a metal isotope in a manner that insures optimal signal intensity with minimal to no signal overlap. Critical considerations include: 1) isotope sensitivity range of the detection instrument, 2) intensity of surface marker expression, 3) degree of variation and patterns of expression across samples, and 4) spillover/background.

1. CyTOF^®^ is most sensitive in the range from atomic mass 153 to 176; therefore isotopes/mass tags within this mass range are preferable for antibodies against weakly expressed markers (51).
2. Surface marker intensity considerations follow a process akin to fluorophore-based panel design for flow cytometry; antigens/probes are first classified as either high expression (primary), medium/variable expression (secondary), or low/unknown expression (tertiary) (81). Antibodies with low binding affinity or directed against tertiary antigens should be paired with isotopes in the detection instrument’s high-sensitivity detection range. Antibodies against primary or secondary antigens need pairing with isotopes on either end of this optimal mass range.
3. The same antigen may have vastly different expression patterns depending on cell type, organ, or disease state (36). For this reason, it is important to either have or obtain knowledge of the specific antigens in the study. Prior knowledge from conventional flow cytometry and literature will help assign antigens to the above-mentioned categories. For example, CD4 is a primary antigen and exhibits a clear bimodal expression, with clear negative and positive populations (82). Alternatively, chemokine receptors such as CCR7 are often classified as secondary antigens and have a broad, often non-modal spectrum of expression (83).
4. Relative to the cellular autofluorescence or channel cross-talk seen in conventional flow cytometry, sources of background are greatly reduced in mass cytometry (20, 36). Mass cytometry background is predominantly caused by signal spillover related to instrument detection sensitivity. In a TOF analyzer ions are separated based on velocity, which in turn is determined by their mass (M) and kinetic energy. Ions of the same kind have small differences in initial position and velocity from each other resulting in slightly different detector arrival times, which is reflected in the width of the resulting mass peak. An over-abundance of the same ions causes position and velocity spreads, resulting in broader mass peaks spilling over into the adjacent mass peak (M+/-1). If an antibody against a high-expressing antigen is conjugated to a metal isotope within the high-sensitivity range of the instrument, spillover will occur due to abundance sensitivity. The second cause of spillover is oxidization of certain metal isotopes following air exposure, resulting in a background signal at 16 mass units (^16^O) higher than the mass of the primary isotope (M+16). Oxide formation can occur in lanthanum (La), praseodymium (Pr), neodymium (Nd), and samanium (Sm)-labelled antibodies/probes. Only 7 metal isotopes form significant oxides: ^139^La, ^142-144^Nd, ^148^Nd, and ^150^Nd. Spillover matrices are available to assist in this process (84). Before sample acquisition, CyTOF^®^ machine parameters are optimized to limit the ^139^La oxidation to less than 3% of ^155^Gd (= M+16). Finally, the largest contribution to signal overspill is isotope impurity, i.e. a contamination of a metal with one of its other isotopes. 100% purity is not pragmatically feasible for all metal isotopes.

The overall principles of panel design (36) are as follows:

1. Tertiary antigens should be paired with isotopes within the CyTOF^®^ high-sensitivity range (85–108) and primary antigens should be paired with isotopes outside this range;
2. Do not place a tertiary antigen-isotope pair in the oxide-mass (M+16) of a primary antigen-isotope pair;
3. Choose isotope tags for tertiary antigens in channels which receive no or little spillover from adjacent channels;
4. For less pure isotopes, select antigens that identify specific cell subsets (for example CD4^+^ and CD8^+^ subsets, which are mutually exclusive outside the gut and thymus);
5. Channels with high spillover can be reserved for markers to be excluded from downstream analysis (for example CD41 to gate out platelets and platelet-cell aggregates).

There are many reference resources that support mass cytometry panel design, including Fluidigm’s online tools, institute mass cytometry core facilities, expert collaborators, and key publications (67). Additionally, available MaxPar panel kits (Fluidigm, San Francisco, CA) (109, 110) include a user-friendly kit-specific data analysis platform (GemStone Software, Topsham, ME).

Although the list of pre-conjugated antibodies for purchase is steadily growing, customized antibody panels often require in-house conjugations. Following panel design, unlabeled antibodies must be conjugated to selected isotopes. Fortunately, conjugation kits and published protocols are available (29, 31, 33–35). The isotope planned for conjugation is first linked to a polymer *via* a chelator. Common chelators used for mass cytometry isotope conjugations include diethylene triamine pentaacetic acid (DTPA), ethylenediamine tertraacetic acid (EDTA), and 1,4,7,10-tetraazacyclododecane-1,4,7,10-tetraacetic acid (DOTA). The antibody is separately modified in its hinge region by a reduction of disulfide bonds to thiols using tris-2-carboxyethyl phosphine (TCEP). Finally, the polymer and associated chelate are coupled to a thiol group of the reduced antibody. These methods can be applied to conjugate metal isotopes to IgG antibodies. Buffers containing protein or glycerol as antibody stabilizers should be avoided. For purified antibodies only available with BSA, BSA removal kits are available that can be used before proceeding to the antibody conjugations (111).

Methods for confirming successful conjugation vary depending on the protocol. This is elegantly outlined by *Han* et al. (29) The shelf life of antibodies conjugated “in-house” can vary significantly. If stored properly they generally maintain functionality at least 6 months from conjugation (29, 112). Antibodies unused/stored for longer periods will require testing on the mass cytometer to confirm that the isotope remains conjugated. How these tests are best performed is explained in the section “*Sample Acquisition & Data Output*”. Once confirmed, the antibody can be used for new cells of interest following standard titration. Results of subsequent titrations should be compared to the initial titration results to identify and troubleshoot new issues.

The next crucial step is to titrate all antibodies and perform a test run involving the entire experimental protocol. This achieves the best possible signal-to-noise ratio by reducing non-specific antibody binding and spillover (113, 114). In addition, using antibodies at non-saturated concentrations prevents ion detector saturation (33). Similar to conventional flow cytometry, a serial dilution strategy of at least 5 dilutions is advisable. Antibodies to primary antigens (e.g. common lineage markers such as CD3, CD19, and CD11b) should be titrated individually and separately. Subsequently, antibodies directed against secondary and tertiary antigens can be titrated within the combined antibody panel, in the presence of the antibodies already optimally titrated against primary antigens. This approach has 3 advantages: 1) to titrate antibodies for staining the population of interest, 2) to enrich the signal by gating cells known to express particular secondary and/or tertiary antigens, and 3) to provide internal positive and negative controls within the titration samples. If titrating for signaling molecules such as cytokines or transcription factors, both a baseline sample and an activated sample (stimulation or treatment) are needed (115). It is advisable to select a cell number per titration point that is comparable to the actual experiment. The antibody titers are determined by calculating the staining index, a method very similar to that used in conventional flow cytometry (116, 117). The main difference between mass cytometry titrations and those used in flow cytometry is that the standard deviation of the negative population is essentially non-existent in mass cytometry titrations and is therefore not included in the staining index formula (118).

Finally, the performance of the panel should be tested on a few control samples prior to proceeding to valuable experimental samples. If the specificity of a signal is unclear, “metal-minus-one” (MMO) controls can be used for resolution (117). If spillover persists with MMO, Chevrier et al. developed a set of computational tools to compensate for spillover in mass cytometry data (see *Compensation*) (84). In some situations, tertiary antigens are so weakly expressed that they are not cleanly discernible. Switching to a 2-step staining can augment the signal from the weakly staining primary antibody; a primary antibody conjugated to biotin, FITC, phycoerythrin (PE), or allophycocyanin (APC) is followed by a metal isotope-labelled secondary antibody or streptavidin. Intelligent mass cytometry panel design is an iterative process often requiring multiple revisions for optimization.

## Sample Staining

Before sample staining, ensure that all buffers are clean by running these solutions at a 1:10,000 dilution (in ddH_2_O or Fluidigm’s cell acquisition solution [CAS]) in the machine’s “solution” mode. This process is further elaborated in “*Sample Acquisition & Data Output*”. Contaminants including barium (Ba) from dish soap used in labware cleaning; or lead (Pb), mercury (Hg) and tin (Sn) from water pipes/distilled water are a common challenge in mass cytometry (**Figure 2A**) (36). An abundance of Ba contacting the detector also damages the detector over time and decreases detector lifespan (51). When working with patient samples, certain therapeutic reagents (cisplatin in cancer chemotherapy, gold in autoimmune therapies), or contrast reagents (Iodine, Ba) can circulate in the patient and contaminate the tissue under study, thus confounding the data (**Figure 2A**) (54, 119). Alternative intercalators are available, including rhodium-103 (^103^Rh) (119).

Samples often require pre-processing prior to staining. For PBMC, the anticoagulant used in the blood collection can affect specific cell types and thereby adversely impact the data (120, 121). It is important to optimize tissue digestion protocols to minimize cell debris (reduces staining quality) and maximize viability. Multiple published protocols exist for a variety of human and mouse tissues, including tumors (43, 57, 61, 122–130). In general, when attempting to enrich cells, protocols should leave the cells of interest unmanipulated (e.g., negative selection procedures for magnetic bead separation or fluorescence-activated cell sorting/FACS). Heavy metals in magnetic beads can interfere with the mass cytometer, so careful washing post-enrichment is required (131). Immune cells from digested tissues can be enriched without antibodies or magnetic beads *via* density gradient isolations using agents such as Percoll^®^ (Cytivia, Marlborough, MA), Ficoll^®^ (Cytivia, Marlborough, MA), and Lympholyte^®^ (Cedarlane, Burlington, LC). Density gradients, can cause differential loss reduced numbers of certain cell types (for example, granulocytes after Ficoll^®^gradient isolations) (132).

When working with cryopreserved samples, the effect of freezing and thawing on target epitopes needs to be tested by conventional flow cytometry prior to initiating a mass cytometry experiment. Freezing can greatly alter surface expression of certain surface antigens (CD62L or PD-1) and cytokines, due to down-regulation under cellular stress (133–135). This can be mitigated by maintaining the samples overnight in cell culture media to allow them to equilibrate following thaw (136). For immune cells, a common media is RPMI with 10% heat-inactivated fetal bovine serum (ΔFBS). To measure cytokine expression potential, cells can be stimulated with phorbol myristate acetate (PMA) (an activator of NF-κb) and ionomycin. To block secretion and thus loss of intracellular signal, at the end of stimulation (2 hours minimum, maximum overnight), treatment with brefeldin A (an inhibitor of protein transport between the endoplasmic reticulum ad the Golgi apparatus) or monensin (an inhibitor of trans-Golgi transport) is required (137–139). Cryopreservation can also differentially affect the relative frequencies of viable subpopulations upon rethaw, and this needs to be optimized to minimize selection bias (140).

When a studying phospho-proteins, because phosphorylation and dephosphorylation is a very rapid and dynamic process, capture of the phospho-protein may require exploratory conventional flow cytometry studies to codify optimal conditions and timing. Samples are often fixed prior to staining, a procedure which must be optimized to minimize selection bias (141–143). It is critical that all antibodies in the panel be confirmed to bind target after fixation, due to epitope denaturation by fixation (144). Occasionally, a new antibody clone must be titrated to replace the clone no longer functional post-fixation. Another approach is to forego sample fixation entirely, and block dephosphorylation by pervanadate incubation for 5 minutes prior to staining (145, 146).

Because mass cytometry target detection relies on antibody Fab’ - target interaction, constant fragment (Fc) receptors need to be blocked prior to antibody staining in order to reduce false positive signal from antibody binding *via* their Fc region (as in conventional flow cytometry) (147). If, however, CD16 (148–153) and CD32 (154, 155) are critical targets, other options include 1) non-specific protein saturation with extra serum or BSA or 2) staining with anti-CD16 and anti-CD32 antibodies in a separate first step, prior to the staining with the remainder of the antibody panel. (The latter approach also leverages competitive inhibition by using the detecting antibodies for simultaneous Fab’-mediated CD16 and CD32 detection and blockade).

Unlike conventional flow cytometry, in which antibody staining is performed at 4°C, mass cytometry staining can be performed at room temperature as internalization of antigens does not alter detection. There are however specific situations in which staining at 4°C is advisable, such as for myeloid populations that adhere to plastic if metabolically active above 4°C or to better preserve cell activation and viability prior to specific functional assays.

After Fc blockade and live sample barcoding, every staining begins with a live/dead cell discernment step using cisplatin. Cisplatin quickly and freely diffuses into dead cells with compromised membranes and forms covalent sulfhydryl bonds with intracellular protein nucleophiles. Cisplatin is commonly applied for live-dead discrimination in mass cytometry because it a) binds covalently to cellular proteins within cells and b) stains cell membranes of viability-compromised cells to a much greater extent than live cells (54). The first property allows cisplatin to remain bound through multiple downstream staining steps used in mass cytometry protocols, and the second property is leveraged for live-dead cell discrimination. Of note, cisplatin needs to be titrated on the sample as diffusion efficiencies vary by tissue (156). This is followed by surface staining (36, 157), after which many parts can be added into staining protocols, such as intracellular (36, 158, 159), intranuclear (160, 161), phospho-staining (141–143), or tetramers (160).

**Figure 3:**
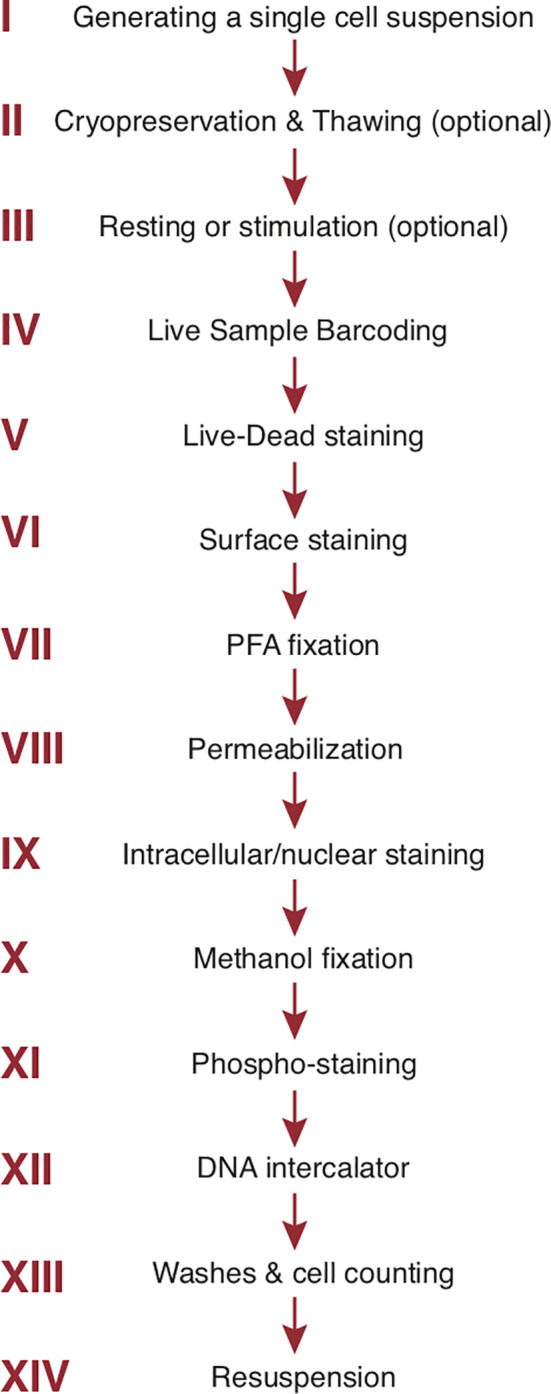
Step-by-step sample preparation. Representative sample preparation protocol. The order of individual steps may vary depending upon the experimental design (see text). Sample washes with cell staining buffer separate each step. The final 3 steps are specific to mass cytometry. *PFA*, paraformaldehyde.

A cryopreservation method has been developed for long-term (9 months) storage of antibody mixes. Use of such a method is highly advisable in large mass cytometry studies with multiple staining cycles to enhance staining reproducibility and data consistency across time (162). The general order of staining steps used by our group is summarized in **Figure 3**. For phospho-protein studies, a dephosphorylation block with pervanadate is inserted at the end of step III (**Figure 3**). The cells are fixed with cold methanol after step IX (intracellular/intranuclear staining) before proceeding to the phospho-staining (step XI) and the subsequent DNA intercalator (step XII) (163). Tetramer staining (e.g., MHC or CD1 tetramers to identify cells with unique TCR specificities) can be incorporated into surface staining (160). Multiple wash steps are incorporated after each staining step to thoroughly remove contamination from unbound reagents and minimize background. As already mentioned, in mass cytometry cells are separated from debris by addition of a DNA intercalator staining incorporated at the end of all staining steps (step XII, **Figure 3**). Natural abundance Iridium (^191^Ir and ^193^Ir) will bind to nucleic acid after cell membranes are permeabilized and the detection of both Ir isotopes allows single cells to be distinguished from debris and doublets (see *Manual Gating*) (7, 164).

Samples are finally washed once with cell staining buffer and counted. Cell numbers need to be kept consistent throughout the experiment and samples need to be resuspended at the optimal cell concentration for sample acquisition, improving data quality and minimizing doublets. The optimal cell concentration depends upon the specific mass cytometry instrument model and its injector type. Each injector has an optimal and maximum event rate, and event rates higher than the maximum indicated for that injector will drastically increase doublets in the resulting data. After any paraformaldehyde (PFA) fixation step, sample staining can be conveniently paused and resumed later by simply storing the sample overnight in 1.6-2% PFA at 4°C. Unlike the case with denaturation of certain conventional fluorophores, metal isotopes are not affected by prolonged fixation steps. If performing phospho-staining, a pause stop can be incorporated with 100% methanol at –80°C. When opting for completing the entire staining protocol in one workday and acquiring the samples on CyTOF^®^ on a subsequent day, samples can be stored in DNA intercalator (e.g. ^191^Ir/^193^Ir cocktail) overnight at 4°C. When storage needs to occur longer than overnight at either of these 2 steps, samples can be washed once with 1 mL cell staining buffer and pellets stored up to one week (165). If stained samples need to be stored longer, *Sumatoh* et al. have developed a method to preserve them in 10% dimethylsulfoxide (DMSO) + 90% FBS at –80°C (165). This long-term storage method was tested on human PBMCs only and therefore may not be applicable for other types of tissues. Our group has confirmed that this protocol also works well for long-term storage of stained mouse splenocytes (*data not shown*).

## Sample Acquisition & Data Output

Sample acquisition on a CyTOF^®^ mass cytometer is performed in one of 2 modes: 1) solution mode or 2) event mode for beads and single-cell analyses. Solution mode is used for testing buffers for contaminants, verifying antibody conjugations, or re-testing older conjugated antibodies (> 6 months). A buffer or conjugated antibody is diluted 10,000 times into either ddH_2_O or CAS and acquired in solution mode. The data output of this type of acquisition includes a mass spectrum showing each element detected as a peak, accompanied by average ion counts. This method does not give the exact number of metal ions conjugated to each antibody. To generate such data, conjugated antibodies must be diluted in Tuning Solution SKU 201072^®^ (Fluidigm, San Francisco, CA), which contains 6 metal isotopes at known concentrations and is used for daily calibration of the instrument. Fluidigm provides users with the necessary protocols and worksheets upon request.

Immediately prior to data acquisition, at least 2 washes with ddH_2_O are required to remove residual buffer salts; these otherwise accumulate at the detector and can cause detrimental instrument drift. Tuning, the process by which the CyTOF^®^ instrument is calibrated before any sample acquisition, maximizes the signal intensity of the metal isotopes within the optimum range from atomic mass 153-176 while minimizing isotope oxide formation (M+16) by the inductively coupled plasma (ICP). The acquisition instrument requires re-tuning every 6 hours during prolonged data acquisition. For more detail on machine tuning and sample acquisition, we refer the reader to helpful Fluidigm machine manuals and a video by Leipold and Maecker (51, 166, 167).

The fluid tubing of a mass cytometer is of much smaller diameter than that of conventional flow cytometers (51). This makes the CyTOF^®^ relatively more prone to obstruction by accumulated debris. For this reason, samples need to be passed over a double 30 mm cell strainer (Partec North America Inc., Swedesboro, NJ) immediately prior to sample acquisition. Even following filtration, there remains a periodic need to unclog the lines during sample runs. Our lab routinely uses BD FACSAria^®^ 50 mm sample inline filters (BD Biosciences, San Jose, CA), which perfectly fit the sample probe line of the Helios^®^.

When using sample barcoding, updating the CyTOF^®^ software with separate labels for all barcodes is strongly recommended throughout the sample run [e.g., for CD45 live barcoding, rather than a uniform label of “CD45”, it is preferable to use “CD45_102” (isotope mass), “CD45_healthy” (experimental group), or “CD45_tumor” (tissue type)]. The importance of this labeling schema is clarified in “*Debarcoding*”.

Importantly, when samples sit in ddH_2_O or CAS, staining intensity and quality declines over time (168, 169) Therefore, samples should be resuspended in these media only immediately prior to data acquisition. Whether ddH_2_O or CAS is the preferred solution for acquisition depends on both the CyTOF^®^ model and injector type associated with that model. For the ball joint injector on CyTOF2^®^ and the narrower HT injector on early Helios^®^ machines [the narrow bore injector (NB)], cell pellets must be resuspended in ddH_2_O. The Helios^®^ injector has a narrower inner diameter, resulting in smaller ion clouds (roughly one half the size of those attained with wide-bore injectors), reducing doublets and doubling the machine’s event acquisition rate (cells/second) (51). However, unintended consequences to the data such as lower median signal intensities and higher coefficients of variation (CVs) have been described using these injectors (170). The Helios^®^ system introduced the wide-bore (WB) injector and CAS to address this pitfall. The WB injector has an inner diameter intermediate between the (narrower) HT injector and the (wider) CyTOF^®^ injector. The CAS has a higher ionic content than water and in combination with the newer WB injector resolves the data quality issues seen with narrow-bore injectors in Helios^®^ datasets (170). One disadvantage of the newer WB injector, however, is that it drastically decreases the maximum event rate from 500 event/sec to 250 events/sec, requiring significantly longer sample acquisition/run times.

During mass cytometry runs, signal drift occurs over time due to gradual accretion of cellular material inside the instrument and associated progressive acquisition delays (51). Signal drift can cause variations between and even within individual data files. Since the sample acquisition speed on a CyTOF^®^ is quite low compared to conventional flow cytometers, experiments are often run across multiple days. The instrument is tuned each day and may be cleaned periodically within a prolonged experiment run, causing additional variance between days. Additionally, in consortia or clinical trials, data is collected at multiple sites and on multiple instruments. To correct for these variances and minimize measurement variations, samples are resuspended in EQ Four Element Calibration Beads^®^ (EQ 4 beads) (Fluidigm, San Francisco). EQ4 beads are polystyrene bead standards containing known relative quantities of metal isotopes from 4 metals (^140^Ce, ^151^Eu, ^153^Eu, ^165^Ho and ^175^Lu), diluted 10-fold in either ddH_2_O or CAS and used for normalizing data within and across experiments (see *Normalization & Concatenation*) (171, 172). This method for minimizing experimental variation is efficient enough that data acquired from different machines can be combined into cumulative datasets. This is important, since different machines have been shown to have discrepancies in their atomic mass sensitivity ranges (173, 174). Recently, Liu et al. further improved the isotopic range of EQ 4 beads by adding 3 elements (^89^Y, ^115^In, and ^209^Bi), resulting in a total of 7 isotopes. The authors demonstrated that the EQ4 beads did not consistently normalize isotope signals outside the mass range of 140-175, whereas the 7-element calibration bead system resolved this issue (175). As of the time of this manuscript publication, 7-element beads are not available for purchase and interested parties must synthesize these themselves.

The output data from the machine is in the form of a Flow Cytometry Standard (.fcs) file (FCS file). The FCS file structure is a standardized array with columns representing channels and rows representing events. This is used for downstream data analysis through programming languages (e.g., R) or FCS file-processing platforms [e.g., FCS Express^®^ (DeNovo Software, Pasadena, CA), Cytobank^®^ (Cytobank Inc, Santa Clara, CA), or FlowJo^®^ (FlowJo LLC, Ashland, OR)]. Generally required items are: 1) the generated FCS file; 2) a panel file listing the metals and corresponding markers; and 3) a metadata file listing the names of all FCS files used, categorizing FCS files into experimental groups (e.g., control, knockout, etc.). The panel and metadata files can be in Comma Separated Values file (CSV) or Microsoft Excel (XSLX) format. Refer to the analysis program/package analysis instructions for guidance in selecting file format.

## Normalization & Concatenation

To correct machine signal variation and thereby minimize measurement variation, “global normalization” (normalization across all FCS files) must be performed. Though this normalization is performed computationally after sample acquisition, it requires the use of bead-based standards added to the sample before sample acquisition and therefore merits advance consideration.

The two algorithms for normalization are **1)** MATLAB^®^-based bead normalization shown by *Finck* et al.(171) and **2)** Fluidigm’s bead identification and normalization (172). The primary difference between these is that MATLAB^®^ normalization compares each file to the other files in the same data set, whereas Fluidigm’s algorithm compares the acquisition files to a set of external values. One pitfall of the Fluidigm algorithm is that the data may be slightly reshaped to fit the external values used as compared to data normalized by the MATLAB^®^ method (176).

In some circumstances, a single sample must be acquired across multiple FCS files and subsequently recombined into a single data file prior to analysis. Common scenarios necessitating such an approach include: 1) a clog occurs in the sample line, sample capillary or the nebulizer requiring the acquisition to be halted and restarted 2) a particularly large sample requiring collection from multiple tubes (168, 169). The recombining of these files into a single FCS file before analysis is termed concatenation (85). Concatenation should be performed in order of file acquisition, to avoid introducing errors from incorrect sequencing (86).

Because events are normalized independently, the order of normalization and concatenation should not affect data quality. However, many algorithms remove artifacts and metadata during the normalization process, which can cause errors during concatenation. For this reason, unless the sample uses a large number of bead standards (> 200 hundred beads), it is advisable to first concatenate and subsequently normalize.

Several different algorithms exist to aid normalization and concatenation. For the MATLAB^®^ algorithm, R packages such as premessa or CATALYST can be used (87, 88). CATALYST fuses all FCS files into a single cell experiment object in R. Therefore if using CATALYST, concatenation is not required. Fluidigm’s algorithmic bead normalization is performed through the instrument’s software. (As of this publication, the software version is CyTOF^®^ 6.7). Note that each batch of EQ 4 beads has its own corresponding passport of external values, which must be updated in the CyTOF^®^ software whenever a new batch of EQ 4 beads is used.

**Figure 4:**
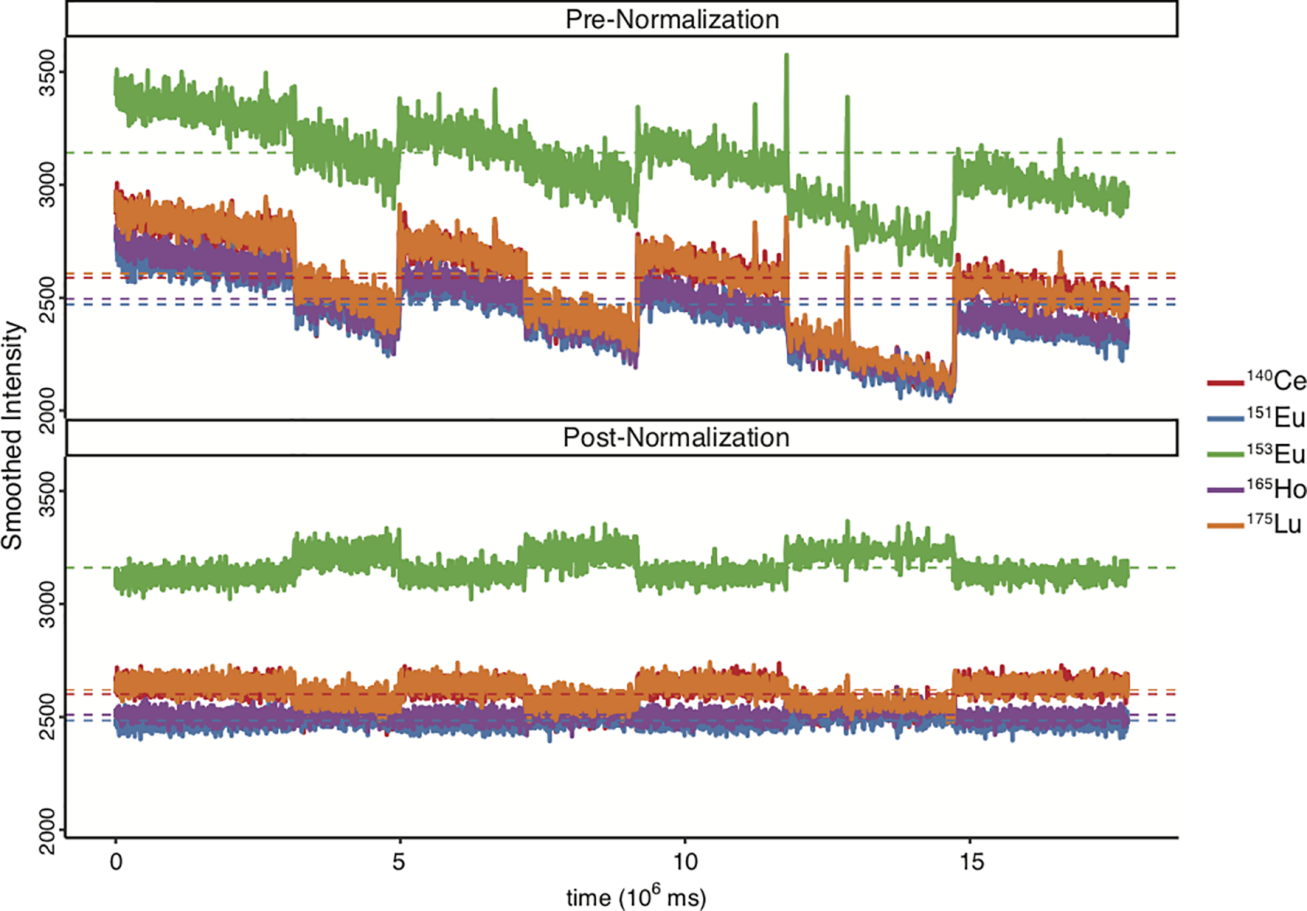
Comparison of bead intensity over time before and after data normalization. *Ce*, Cesium; *Eu*, Europium; *Ho*, Holmium; *Lu*, Lutetium. EQ4 beads were used during sample acquisition. Figure was generated in R *via* the CATALYST package using their standard settings.

Though doublets have historically been considered noise or unwanted confounders, *Burel* et al. found that the molecular signature of T-cells in complex with other immunological cells such as monocytes or B-cells showed phenotypic differences when compared to unattached single T-cells. Moreover, they observed an increase of T-B-cell complexes in the circulation of type-1 diabetes patients (89). The implication of these findings is that removal of doublets may in certain studies skew the data in ways that miss important biological outcomes. However, since most doublets are indeed unwanted noise, it is generally advised to study doublets separately from the singlet data analysis to determine their value to any individual experiment, to avoid compromising experimental rigor. A good QC step to visualize the effect of normalization is to plot the bead intensity over time before and after normalization (**Figure 4**).

## Debarcoding

In barcoding, each sample is labeled with a unique identifier and samples are mixed in a single tube before data acquisition. The output is a single FCS file consisting of the multiplexed data from all of the barcoded samples in the tube. Therefore, an extra step must be taken following data acquisition to split the output data from each barcode into its own FCS file. This process is termed “debarcoding”.

In debarcoding, events are stored in new FCS files corresponding to the individual barcodes, identified by the debarcoding algorithm. The number of output FCS files is equal to number of barcoded samples used. Samples are re-assigned a unique identifier in the debarcoding process. During debarcoding, the single-cell deconvolution algorithm must define which channels are positive and negative for each cell. For this, a threshold value is selected, and cells above this threshold value will be assigned to their corresponding barcode sample (90). The threshold can be selected manually or with the help of an algorithm (e.g. CATALYST provides estimation of threshold values). Rarely, high stringency in such parameters can reduce overall event number in the dataset.

The vast majority of unassigned events are cell-cell doublets or debris (33). Occasionally, cells can be sorted into incorrect barcode channels (false assignment). This false assignment rate is generally under 0.5% under uniform sample staining/experimental conditions. A valuable internal control is to leave 1 barcode from the scheme unused, allowing an estimation of the false assignment rate for debarcoding.

Both premessa and CATALYST packages in R can be used to debarcode (88, 91). Debarcoding can also be done using the MATLAB Compiler Runtime (33). Graphical User Interfaces (GUIs) are available for these processes. GUIs are pre-programmed interfaces that run through the code, allowing analysis without directly interacting with the code. Most of these algorithms require a debarcoding scheme alongside the FCS file, typically in the form of a table that correlates the sample identity to the element mass of the barcode isotope. This instructs the algorithm which barcode corresponds to a sample’s FCS file. Formatting varies by the debarcoding resource.

## Compensation

After normalization, concatenation and debarcoding, compensation is generally the final data pre-processing step in mass cytometry workflow. Compensation is the process whereby detection signal spillover is resolved. In conventional flow cytometry, this is a routine practice due to overlapping excitation and emission spectra of fluorophores and spillover which correlates linearly with fluorophore signal intensity (92). Compensation subtracts the percentage of a fluorophore’s spillover from the measured signal in that particular channel.

Mass cytometry can have spillover, but antibody titrations and intelligent panel design can optimize the signal-to-noise ratio such that compensation is no longer necessary (67). *Chevrier* et al. performed a detailed analysis and found that mass cytometry spillover has a similar linear relationship with the primary signal (84). This relationship has been applied to generate a spillover matrix allowing compensation to be performed with the CATALYST package either in R or *via* a web application created by the same group (87, 93). Another helpful algorithm is CytoSpill, a statistical program that aims to minimize spillover effects. Unlike the CATALYST algorithm or conventional flow cytometry analysis programs, this algorithm does not require the use of single-cell controls, making the process significantly cheaper and easier (94, 95).

## Manual Gating

During the experiment, samples have been put through various stresses which results in the events in the FCS file being a combination of a) live cells, b) dead cells, c) cell-cell doublets, d) beads e) cell-bead doublets and f) bead-bead doublets. The goal of this step is to purge the data as much as possible of b-f, such that the output consists of live single cells. Manual gating can be performed using many established platforms from conventional flow cytometry, such as FlowJo^®^ or FCS Express^®^. Though usually done *via* manual gating, some algorithmic methods such as that in the CATALYST pipeline can efficiently remove d-f.

**Figure 5:**
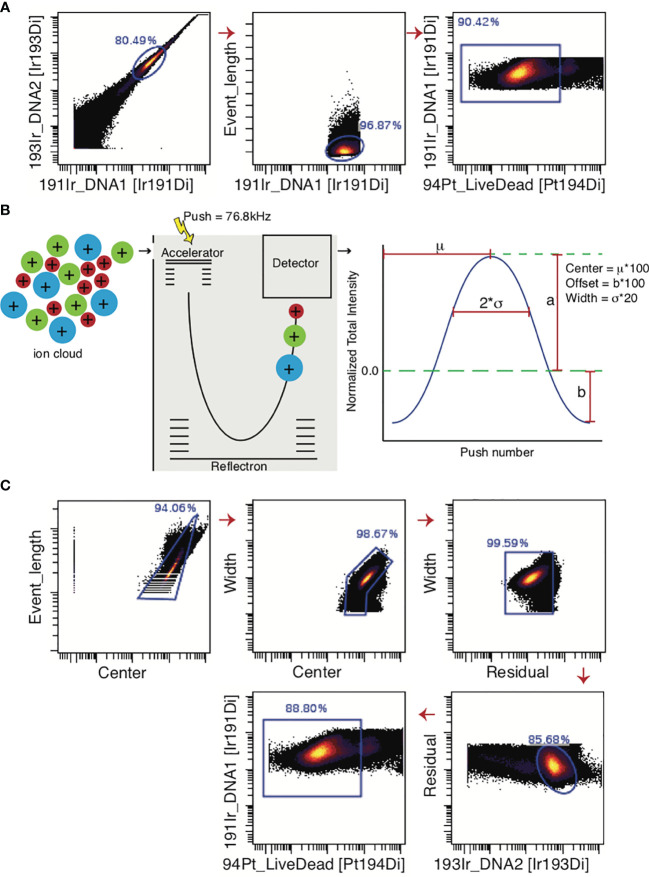
Gating strategies for live single cell events. **(A)** For data generated by the CyTOF2^®^ machine, the predecessor of Helios^®^, single cells were gated only on the bases of Event Length and the DNA intercalator. Live cells are selected as cisplatin-negative events. **(B)** In the Helios^®^ machine, the Gaussian parameters are generated in the Time-Of-Flight chamber where ions are separated on mass-to-charge ratio. Once these ions encounter the detector, an electronic pulse is generated, which is mathematically smoothened into a bell curve. Center, Offset, and Width can be extracted from this curve. **(C)** The added Gaussian parameters greatly improve the ability to select a single-cell population. Note that if beads are not excluded through the normalization process, they must be gated out manually before gating for single live cells.

In **Figure 5A**, a standard mass cytometry gating strategy is shown. Here, the “event length” parameter is used in conjunction with a DNA intercalator to select single cells, followed by live/dead gating based upon cisplatin intercalation. Though single-cell gating using this method has proven difficult, the Helios^®^ mass cytometer is now equipped with additional Gaussian parameters (e.g. center, offset, width, residual), which can be used to streamline data (96). These parameters are generated in the TOF chamber. The ions enter the TOF chamber *via* a narrow slit (**Figure 5B**). Every 13 µs (exact interval varying by machine), a high-voltage pulse provides equal energy to all ions that have accumulated in the chamber during the interval, accelerating them across the TOF chamber and onto the detector. This high-voltage pulse is a push. As the energy provided to the ions is uniform, velocity varies by mass and ions with greater mass require longer times to reach the detector. Once these ions hit the detector, they generate an electronic pulse which when plotted and mathematically smoothened takes the shape of a bell curve (Gaussian distribution) from which the Gaussian discrimination parameters (center, offset, and width) are extracted (**Figure 5B**) (51, 97). The Residual parameter is extracted by calculating the difference between the actual electronic pulse plot and the smoothened bell curve (97). The improved gating strategy is shown in **Figure 5C**.

## Understanding Data Quality

**Figure 6:**
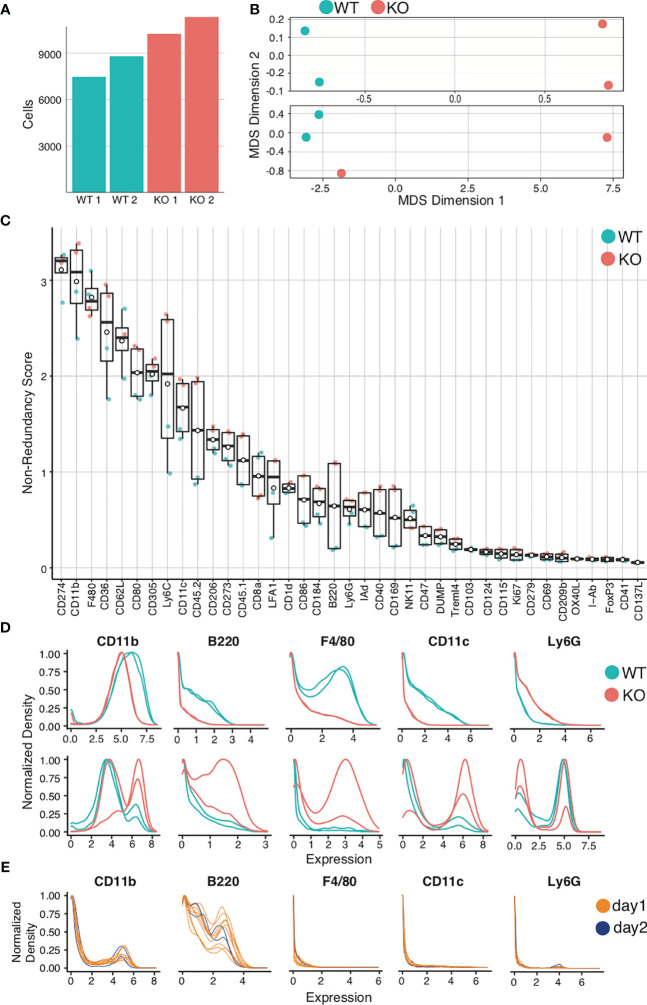
Quality control and marker selection. Mouse bone marrow-derived dendritic cells were generated. Quality control and marker selection was performed on debarcoded/cleaned FCS output after gating to select for live dendritic cell singlets. Figures were generated in R *via* the CATALYST^®^ package using their standard settings. **(A)** Absolute cell number comparisons for a representative analysis using wild-type (WT) and a particular strain of monogenic knockout (KO) mice. **(B)** Multidimensional Scaling (MDS) plots comparing mass cytometry runs from 2 WT and 2 KO samples are shown **(A, C)** Non-Redundancy Score (NRS) plot listing the variation between each sample for each marker in the CyTOF panel. **(D)** Histogram comparing intensity of marker expressions between the samples (Marker Expression Distribution). Due to different sample sizes and distributions, the data was normalized between 0 and 1. **(E)** Histogram demonstrating the use of spiked-in internal control samples across multiple tubes and data acquisition days.

Once the runs are pre-processed, all events are expected to represent live single cells. Before proceeding, it is worthwhile to visualize the number of cells in each sample. This can be done in R with a simple bar graph visualizing the cell number within each sample and comparing across samples (**Figure 6A**). If the cell count is insufficient and the population of interest is a very rare or marginal proportion of the total cells (expected to vary by experimental conditions or cell/tissue type), the experiment may have to be rerun. Even if sufficient cells exist in the input samples, if the difference in cell numbers between runs is extremely large, such variability can affect the statistical analysis. In such cases, the cells can be randomly subsampled (randomly selected from the experimental samples based on parameter matching to controls) or the experiment repeated ensuring larger cell counts.

The next step is to visualize relationships between samples. One cumulative data visualization method is Multidimensional Scaling (MDS) (98). In the sample MDS plot showing data for murine bone-marrow derived dendritic cells (BMDCs) (**Figure 6B**, *upper panel*), the controls and comparisons form distinct groups, indicating that wild-type (WT) mice are similar to one another and knockout (KO) mice are similar to one another, but that there is a significant difference between WT and KO mice. In the lower MDS plot (**Figure 6B**, *lower panel*), while the WT are similar and form a distinct group, the KO mice are dissimilar. Furthermore, one KO clusters with the WT controls. This should prompt evaluation for experimental errors requiring re-analysis. Errors can arise from variances in staining, runs on distinct days, and human error, which can be challenging to troubleshoot. For this reason, we recommend adding a barcoded internal control in each tube as a quality control (“spiking”). Errors can be identified by variation of internal controls from expected outcomes.

After critically assessing cell count and sample quality, one may proceed to analyze the specific markers for the experiment, beginning with a Non-Redundancy Score plot (NRS plot) (**Figure 6C**) (99). This graph visually represents the variation across all samples by individual marker. Ideally, the marker variation is caused by the biologic differences between experimental groups. However, large variations in cell count or in staining intensity can also be sources of variation on the NRS plot. Markers that contribute minimal or no variation between samples can be excluded from clustering. To view the marker expression in more detail, a histogram can be used to visualize the results (**Figure 6D**). Marker expression histograms are extremely useful when plotting an internal control sample spiked into a tube (by barcoding). This provides a quality control for staining reproducibility. In **Figure 6E**, the internal controls are color-coded by day of staining, demonstrating that reproducibility was not optimal.

## Data Transformation

After bead normalization, data must be transformed to allow proper distinction between cell populations positive and negative for each marker. The most common method is the arcsinh transformation, using a cofactor of 5 (99). In R, CATALYST packages have functions that can perform this transformation (99, 100). This transformation can also be accomplished directly in Cytobank^®^ or FlowJo^®^; however, this is lost when exporting.

## Clustering

The next step is data clustering based on group similarities between cells. This data visualization method compartmentalizes events (cells) into groups which are then used to insure the detection, characterization, and calculation of relative abundance of the different populations in the sample. Note that not all clusters/groups represent a cell population, as some clusters might represent the same population with slightly differing marker expression. For this reason, it is important to carefully study and compare the marker expression within each cluster. Several different clustering algorithms are available for mass cytometry data, differing primarily based on the criteria they use to assign a cluster designation. The highest performing algorithms are FlowSOM, X-Shift and PhenoGraph (101). If specifically looking for rare populations, X-Shift is preferable due to its statistical power to detect rare events (102).

While the algorithms themselves use a variety of models to cluster the data, they utilize the marker expression within each individual cell to distinguish the clusters based on phenotype. Therefore, it is important to determine in advance which cells to isolate and the potential cell types present in the samples, and design the experimental panel accordingly. The more distinguishing markers in the panel (markers that can be used to show a clear and meaningful difference in the subpopulations in the sample), the better the quality of the clustering will be to allow clear differentiation and ease of population identification (103). As explained in detail in the previous section (*Understanding Data Quality*), an NRS plot (**Figure 6C**) is utilized in order to select the markers most relevant for clustering; meaning markers with a low non-redundancy score will not contribute to the cluster outcome and can therefore be excluded from clustering analysis. The clustering algorithms use the data on event phenotype (using the markers selected by the analyst) to group events into clusters or “bins”. Clustering is generally performed over all FCS files to allow sample/file comparability in downstream analyses. R or Python are the recommended clustering programs, as these allow much greater control over the statistical binning process. Packages such as CATALYST or Phenograph can be used, the details of which can be found in their respective workflows (99, 104). Note that apparently different clustering packages or software might ultimately utilize similar algorithms. For example, CATALYST uses the FlowSOM algorithm for clustering (105, 106). Thus, it is important to study in advance the workflow being used and identify which algorithms may best suit the output data, well in advance of the experimental run.

**Figure 7:**
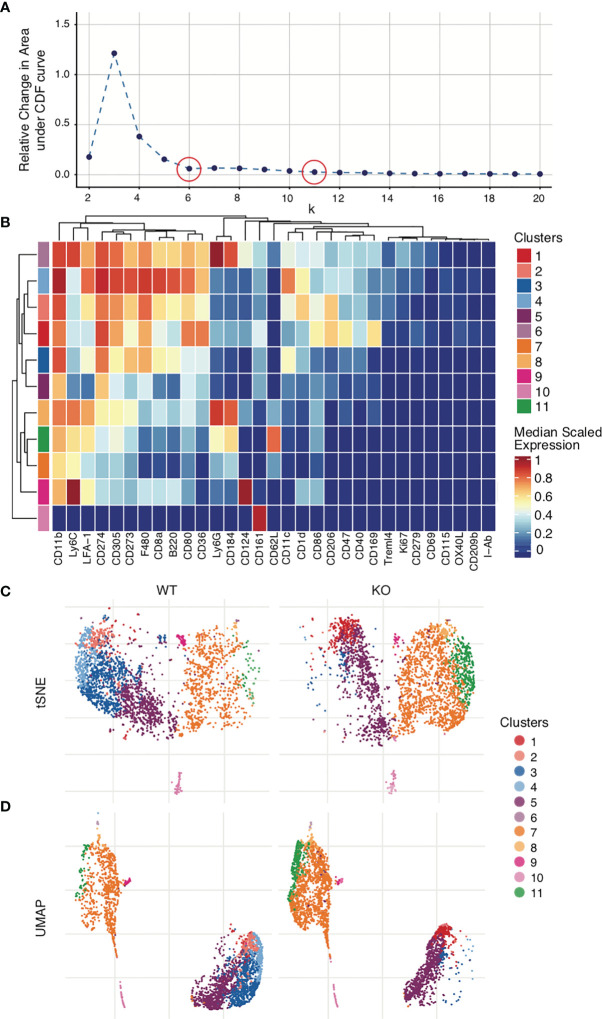
Clustering and visualization. Mouse bone marrow-derived dendritic cells were generated. Clustering was performed on live dendritic cell singlets after Quality control and marker selection. Figures were generated in R *via* the CATALYST package using their standard settings. **(A)** Deltaplot showing relative change under the Cumulative Distribution Function (CDF) when clustering is performed using different cluster numbers. The optimal cluster number is indicated at the threshold below which a change in cluster number no longer correlates to a change under the CDF (plateau point in the graph). *red circles*, *selected cluster values.* **(B)** Clusters have been created across all FCS files. Expression levels for each marker are normalized across all clusters. The heatmap shows the normalized expression levels of each marker in each cluster in the total data set. The upper legend on the right of the heatmap indicates the clusters, coded by color to the corresponding row indicated on the far left of the heat map. The number of clusters used in the analysis was selected by deltaplot **(A)**. Markers were selected based on the NRS plot (**Figure 6C**). (C) tSNE comparison of WT vs KO samples, demonstrating clusters 3 and 4 severely diminished and cluster 11 greatly increased in KO as compared to WT. **(D)** UMAP comparison of WT vs KO.

While some algorithms such as PhenoGraph will automatically select the optimum number of clusters to use, others like FlowSOM will require manually selection of the cluster number (k) (104, 105). One method for manual selection is initial generation of a large number of clusters (~100), followed by serial merging of particular clusters until the optimum cluster number is obtained. Again, not every cluster represents a subpopulation in the sample, and it is here that applying biological understanding is important. Whether these clusters represent a novel/separate subpopulation (creating a new cluster) or are similar to each other and therefore will need to be merged must be determined based on the marker expression levels on the different clusters. All clusters that represent a will be continually merged until only clusters that are determined to be biologically different from each other remain. The same process can be performed statistically with the help of a delta plot, where the relative change under the Cumulative Distribution Function (CDF) is plotted for each cluster value. It is no longer optimal to increase the number of clusters selected (k) when doing so results in no/minimal change in the CDF (**Figure 7A**). A delta plot is generated using either the ‘deltaPlotR’ Package or the CATALYST package (107, 108). When clustering initiates, a randomly generated ‘seed value’ is used as the starting point of the clusters. To ensure that clustering is reproducible, one should record the seed value used to initiate clustering (177). To ensure that clustering is robust and to avoid the possibility of error due to random selection of seed value, clustering should be repeated with at least 3 random seed values and output clusters compared. These should approximate each other closely.

A good QC measure to ensure clustering has been correctly performed is to include markers for a known control cell population within the sample. When opting for internal controls, clustering could be performed on the FCS files separately in order to verify similar cluster proportions between tubes.

## Visualization and Interpretation

The next step is to determine whether clusters represent distinct populations and to phenotype each cluster based on statistical analysis and graphical outputs. A combination of biological and statistical expertise become highly relevant at this stage. R packages like FlowSOM, FlowCore and CATALYST offer several different visualization functions. The most common visualization approaches are heatmaps. As seen in **Figure 7B**, the expression for each marker is aggregated by cluster. These clusters were generated from FCS files and therefore can be applied to any group used in the analysis (in this case, WT murine controls and knockouts). Heatmaps not only help to visualize markers expressed in different clusters on the merged FCS files, but also to estimate the relative expression intensity. Marker expression levels determine to which immune lineages these clusters may belong, elucidate novel cell clusters or phenotypes, and can aid in determining whether certain clusters can be merged. The heatmap parameters can be adjusted depending on use case (e.g., comparing a particular marker’s expression across groups rather than comparing prevalence of clusters, etc) (178, 179).

Mass cytometry data is of necessity multi-dimensional. Clusters, marker intensity, and differing markers complicate interpretation. To address this, dimensionality reduction algorithms can be applied to concisely visualize multiparametric data in two dimensions. t-distributed Stochastic Neighbor Embedding (tSNE), Principal Component Analysis (PCA), Isometric Feature Mapping (Isomap), and Uniform Manifold Approximation and Projection (UMAP) are all common methods for high-dimensional data visualization (99, 180–183). These facilitate easy identification of the experimental group differences by visually organizing populations using distance on the plot as a surrogate for similarity between groups. The most commonly used approaches are tSNE and UMAP. UMAP, unlike tSNE, is primarily non-parametric nonlinear dimensionality reduction algorithm originally designed to preserve global data structure with smaller data sets, though more recently parametric UMAP was demonstrated to compare favorably in performance to its on-parametric counterpart while adding the benefit of learned mapping of new data. In UMAP, distances both within and between clusters are generally meaningful; whereas in tSNE, distances between clusters may not be. Therefore, UMAP is the preferred mode of visualization (181, 184). In **Figures 7C, D**, a case is presented in which both tSNE and UMAP efficiently visualize the cell clusters present in the WT control and KO mouse groups. Figures have been generated in R *via* the CATALYST package using their standard settings. One can also use Flow Core in R, or directly generate such visualizations through Cytobank.

Once clusters have been visualized and characterized, they can be annotated in R. Clustering can require hours to days to complete depending on the dimensionality reduction algorithms and the acquired total cell count per group. To prevent prolonged run times, these algorithms are ideally run on a subsample of the total population (~2000 cells). When looking for a particularly rare population, more cells can be selected for algorithm application. Because subsampling is random, a set seed function in R is used to ensure reproducibility. To ensure clusters are not lost in the sample and that subsamples are representative of the total sample, at least 3 iterations should be performed with 3 different seed values to confirm similar results (185).

Another method to visualize the relationship between clusters is the diffusion map. Clusters are arranged in order of overall similarity, creating the appearance of a gradient based on change in marker intensity per analyzed event. Diffusion maps are commonly used to study the differentiation or lineage origin of individual cells in a sample set. Another useful application of this visualization technique is for groups of cells of the same type, taken at different time points in treatment or maturation of a sample (186). When viewing diffusion maps, it is critical to remember that association does not imply causality. Viewing 2 adjacent clusters in a diffusion map indicates that they are the most similar in the sample, but not necessarily that one has differentiated from the other. Data analytics must be tempered with biological understanding of the cell type. An algorithm commonly used for this type of analysis is Wanderlust (187). Wanderlust assumes a linear trajectory and has significant limitations with complex datasets or in the case of multiple cell fates. For those datasets with potential bifurcating branches, Wishbone or Monocle 2 can be applied (188, 189).

There are several other resources that can be applied in place of or in addition to R coding such as FlowJo^®^, FCSExpress^®^, and Cytobank^®^ (190–192). Several R scripts also offer GUIs making it significantly easier to analyze such data (e.g., Cytofkit package for Phenograph). However, R allows the maximum ability to fine-tune parameters, making it extremely attractive as an analysis platform (193). Finally, Astrolabe (Astrolabe Diagnostics, Fort Lee, NJ) offers an interactive platform in which the researchers can interact directly with a computational biologist to analyze data (194). Recently, automatic annotation algorithms have started to be developed [e.g., Automated Cell-type Discovery and Classification (ACDC)], which allows users to input biological parameters such as marker expression levels and apply this to identify such populations within the sample (195).

## Limitations

Currently, the sensitivity of metal isotope-tagged antibodies is lower than that of the most quantum-efficient fluorophores, such as phycoerythrin (PE). The main reason for this is that chelating polymers used for antibody conjugation can only accommodate a maximum of 100 metal ions, creating a ceiling on signal intensities and making it more difficult to measure extremely weakly expressed markers (20, 21). Another major drawback of mass cytometry is the much lower acquisition flow rate as compared to flow cytometry. This is due to the dynamics in the TOF chamber, resulting in longer acquisition times. Where conventional flow cytometers can have a flow rate of up to 50,000 events/second, CyTOF2^®^ only has a flow rate of 500 events/second (250 events/second for Helios^®^) (167, 196). Despite these low flow rates, mass cytometry machines are also quite prone to clogging due to the small diameters of the sample lines and nebulizer. The latter is easily resolved by passing samples through a cell strainer both before and during sample acquisition as explained in “*Sample Acquisition & Data Output*”. The latest CyTOF XT^®^ machine from Fluidigm is capable of sensing and removing clogs automatically. In addition, sample preparation and staining require extra caution with regards to possible heavy metal contaminants such as barium from dish soap or lead from distilled water. Another limitation is the high cost of metal-tagged antibodies, conjugation kits, and other reagents. Although the costs are significantly higher as compared to flow cytometry, these are still much lower than for scRNAseq (21). With increasing number of users and advancements in reagents and technology, these costs are expected to decrease. Finally, because cells are vaporized during the analysis, sorting populations of interest is not currently possible with mass cytometry. To address the latter, one can sort populations on a fluorescent-activated cell sorter for follow-up studies.

The increased number of parameters made available by mass cytometry complicates data analysis, requiring improved bioinformatics approaches for accurate interpretation and visualization of fcs files. Data analytics requires a combination of biological, statistical, and programming knowledge, making the overall process tedious for single individuals. Though packages like ParkerICI/premessa and CATALYST in R are designed to make analysis significantly more user-friendly (88, 93), without significant R programming skills, the researcher remains largely restricted to package workflow and graphing programs. More recently new options have arisen including GUIs and web interfaces that allow analysis without necessitating R programming skills. Most notable among these is Cytobank^®^ (192).

Another potential limitation during manual cluster analysis is the potential for operator bias. This can be significantly mitigated by automating cluster identification through algorithms such as CITRUS (Cluster Identification, Characterization, and Regression) (197, 198). CITRUS automatically clusters cells in each experimental group and compares them based on inputted biological parameters as well as data from publicly available datasets.

## Future Directions

Despite rapid technological advancements and vast capacities, mass cytometry has many areas ripe for future development. At the instrument level, increasing the flow rate (reducing time of analysis) without losing data quality or altering machine sensitivity are key needs. The newest mass spectrometers, have a more sensitive detector and/or improved TOF chambers than prior Helios^®^ mass cytometers (51, 199, 200). Novel polymer chelators able to accommodate more than 100 metal ions at a time could increase signal intensities significantly and thus empower investigations of very weakly expressed molecules (tertiary antigens). Additional isotopes and new methods to purify existing isotopes at a requisite (>98%) purity and novel conjugation chemistries will allow mass cytometry to approach the theoretical 120-parameter capabilities of the Helios^®^ (**Figure 2A**). With the recent development of compensation tools, stringency requirements for metal isotope purity may be relaxed (84, 95). By merging multiple mass cytometry panels into a single clustering analysis, the computational tool CyTOFmerge is greatly expanding the number of simultaneously analyzable parameters (201).

Annotation is one of the most difficult portions of any exploratory mass cytometry analysis. It requires a depth of biological knowledge regarding the unknown cell population, which the researcher may lack at the time of the experiment. One solution is to compare the experimental dataset to available reference datasets. This approach is seen in techniques such as Seurat, a pipeline for scRNAseq. These reference maps provide information about cellular phenotype and interactions, creating a framework to compare existing data with unknown cell populations to aid in their identification and classification. Scaffold is another example of a system for immune cells, built on flow and mass cytometry data sets. Unlike for genomic data, such reference maps are scarce for mass cytometry and represent invaluable resources for future development (202).

Another much-needed application in mass cytometry is a method similar to pseudotemporal ordering used in scRNAseq. This would support immune lineage differentiation studies, allowing clusters originating from common lineages to be identified and mapped. However, distinct from pseudotemporal ordering in scRNAseq, which uses transcription data for its analysis, this would require the use of lineage markers and other characteristic surface proteins (203).

Ultimately, the greatest limitation to more widespread application of mass cytometry is the difficulty in post-acquisition data analysis and interpretation. Automatic screening and comparison of mass cytometry output variables through databases and existing publications represents a major step forward in understanding and annotating cells. Artificial Intelligence (AI) systems are likely to become standards in the near future to make increasingly accurate predictions and annotations based on the ever-increasing available data (204, 205). Automating the entire pipeline into a one-click analysis, with selectable output visualizations and analysis factors, will make the technique more accessible for wider applications of mass cytometry in such key expanding areas as TME responses and immunotherapies.

## Author Contributions

AI, AH, and AP performed the literature compilation, writing, editing, and proofreading. All authors contributed to the article and approved the submitted version.

## Funding

Funding support to the authors was provided by 1R01HL133462 (NIH) (AH, AP) and the Batchelor Foundation for Pediatric Research (AH, AP).

## Conflict of Interest

The authors declare that the research was conducted in the absence of any commercial or financial relationships that could be construed as a potential conflict of interest.

## Publisher’s Note

All claims expressed in this article are solely those of the authors and do not necessarily represent those of their affiliated organizations, or those of the publisher, the editors and the reviewers. Any product that may be evaluated in this article, or claim that may be made by its manufacturer, is not guaranteed or endorsed by the publisher.

## References

[1] Bandura DR Baranov VI Ornatsky OI Antonov A Kinach R Lou X. Mass Cytometry: Technique for Real Time Single Cell Multitarget Immunoassay Based on Inductively Coupled Plasma Time-of-Flight Mass Spectrometry. Anal Chem (2009) 81(16):6813–22. doi: 10.1021/ac901049w 19601617

[2] Stern L McGuire H Avdic S Rizzetto S Fazekas de St Groth B Luciani F. Mass Cytometry for the Assessment of Immune Reconstitution After Hematopoietic Stem Cell Transplantation. Front Immunol (2018) 9:1672. doi: 10.3389/fimmu.2018.01672 30093901 PMC6070614

[3] McGuire HM Rizzetto S Withers BP Clancy LE Avdic S Stern L. Mass Cytometry Reveals Immune Signatures Associated With Cytomegalovirus (CMV) Control in Recipients of Allogeneic Haemopoietic Stem Cell Transplant and CMV-Specific T Cells. Clin Transl Immunol (2020) 9(7):e1149. doi: 10.1002/cti2.1149 PMC7332355 32642063

[4] Aries J Charrot S Ball J Mee M Theocharidis S Van Gassen S. Integrated Immune Signature Analyses Identifies Evolution of Distinct Immunoregulatory Cell Populations Which Control Alloreactivity After Allogeneic HSCT. Blood (2019) 134(Supplement_1):595. doi: 10.1182/blood-2019-124875

[5] Matos TR Hirakawa M Alho AC Neleman L Graca L Ritz J. Maturation and Phenotypic Heterogeneity of Human CD4+ Regulatory T Cells From Birth to Adulthood and After Allogeneic Stem Cell Transplantation. Front Immunol (2021) 11:3447. doi: 10.3389/fimmu.2020.570550 PMC7848157 33537026

[6] Hirakawa M Matos TR Liu H Koreth J Kim HT Paul NE. Low-Dose IL-2 Selectively Activates Subsets of CD4+ Tregs and NK Cells. JCI Insight (2016) 1(18):e89278. doi: 10.1172/jci.insight.89278 27812545 PMC5085610

[7] Gadalla R Noamani B MacLeod BL Dickson RJ Guo M Xu W. Validation of CyTOF Against Flow Cytometry for Immunological Studies and Monitoring of Human Cancer Clinical Trials. Front Oncol (2019) 9:415. doi: 10.3389/fonc.2019.00415 31165047 PMC6534060

[8] Levine JH Simonds EF Bendall SC Davis KL Amir ED Tadmor MD. Data-Driven Phenotypic Dissection of AML Reveals Progenitor-Like Cells That Correlate With Prognosis. Cell (2015) 162(1):184–97. doi: 10.1016/j.cell.2015.05.047 PMC4508757 26095251

[9] Krieg C Nowicka M Guglietta S Schindler S Hartmann FJ Weber LM. High-Dimensional Single-Cell Analysis Predicts Response to Anti-PD-1 Immunotherapy. Nat Med (2018) 24(2):144–53. doi: 10.1038/nm.4466 29309059

[10] Maby P Corneau A Galon J. Phenotyping of Tumor Infiltrating Immune Cells Using Mass-Cytometry (CyTOF). Methods Enzymol (2020) 632:339–68. doi: 10.1016/bs.mie.2019.07.025 32000904

[11] Lavin Y Kobayashi S Leader A Amir E-AD Elefant N Bigenwald C. Innate Immune Landscape in Early Lung Adenocarcinoma by Paired Single-Cell Analyses. Cell (2017) 169(4):750–65.e17. doi: 10.1016/j.cell.2017.04.014 28475900 PMC5737939

[12] Gonzalez VD Samusik N Chen TJ Savig ES Aghaeepour N Quigley DA. Commonly Occurring Cell Subsets in High-Grade Serous Ovarian Tumors Identified by Single-Cell Mass Cytometry. Cell Rep (2018) 22(7):1875–88. doi: 10.1016/j.celrep.2018.01.053 PMC8556706 29444438

[13] Roussel M Lhomme F Roe CE Bartkowiak T Gravelle P Laurent C. Mass Cytometry Defines Distinct Immune Profile in Germinal Center B-Cell Lymphomas. Cancer Immunol Immunother CII (2020) 69(3):407–20. doi: 10.1007/s00262-019-02464-z PMC7764565 31919622

[14] Lowther DE Goods BA Lucca LE Lerner BA Raddassi K van Dijk D. PD-1 Marks Dysfunctional Regulatory T Cells in Malignant Gliomas. JCI Insight (2016) 1(5):e85935. doi: 10.1172/jci.insight.85935 27182555 PMC4864991

[15] Abel B Fehlings M Nardin A Newell E Yadav M. Immuno-Phenotyping of Tumor-Specific CD8 T Cells Using High-Dimensional Mass Cytometry. J Immunol (2020) 204(1 Supplement):86.7–7. doi: 10.1158/2326-6074.tumimm19-a8

[16] Hartmann FJ Babdor J Gherardini PF Amir E-AD Jones K Sahaf B. Comprehensive Immune Monitoring of Clinical Trials to Advance Human Immunotherapy. Cell Rep (2019) 28(3):819–31.e4. doi: 10.1016/j.celrep.2019.06.049 31315057 PMC6656694

[17] Herbrich S Cavazos A Cheung CMC Alexander-Williams L Short NJ Matthews J. Single-Cell Mass Cytometry Identifies Mechanisms of Resistance to Immunotherapy in AML. Blood (2019) 134(Supplement_1):1428. doi: 10.1182/blood-2019-128601

[18] Nolan JP Condello D. CHAPTER: Unit1.27. In: Spectral Flow Cytometry, Robinson JP, Ed Al M, eds. Curr Protoc Cytomz (2013). New Jersey, United States: Wiley–Liss Hoboken, New Jersey, United States.

[19] Park LM Lannigan J Jaimes MC. OMIP-069: Forty-Color Full Spectrum Flow Cytometry Panel for Deep Immunophenotyping of Major Cell Subsets in Human Peripheral Blood. Cytometry A (2020) 97(10):1044–51. doi: 10.1002/cyto.a.24213 PMC8132182 32830910

[20] Bendall SC Nolan GP Roederer M Chattopadhyay PK. A Deep Profiler’s Guide to Cytometry. Trends Immunol (2012) 33(7):323–32. doi: 10.1016/j.it.2012.02.010 PMC3383392 22476049

[21] Spitzer MH Nolan GP. Mass Cytometry: Single Cells, Many Features. Cell (2016) 165(4):780–91. doi: 10.1016/j.cell.2016.04.019 PMC4860251 27153492

[22] Kashima Y Togashi Y Fukuoka S Kamada T Irie T Suzuki A. Potentiality of Multiple Modalities for Single-Cell Analyses to Evaluate the Tumor Microenvironment in Clinical Specimens. Sci Rep (2021) 11(1):341. doi: 10.1038/s41598-020-79385-w 33431933 PMC7801605

[23] Maecker HT Trotter J. Flow Cytometry Controls, Instrument Setup, and the Determination of Positivity. Cytom Part J Int Soc Anal Cytol (2006) 69(9):1037–42. doi: 10.1002/cyto.a.20333 16888771

[24] Maecker HT Frey T Nomura LE Trotter J. Selecting Fluorochrome Conjugates for Maximum Sensitivity. Cytometry A (2004) 62A(2):169–73. doi: 10.1002/cyto.a.20092 15536642

[25] Futamura K Sekino M Hata A Ikebuchi R Nakanishi Y Egawa G. Novel Full-Spectral Flow Cytometry With Multiple Spectrally-Adjacent Fluorescent Proteins and Fluorochromes and Visualization of *In Vivo* Cellular Movement. Cytom Part J Int Soc Anal Cytol (2015) 87(9):830–42. doi: 10.1002/cyto.a.22725 PMC5132038 26217952

[26] Cossarizza A Chang H-D Radbruch A Akdis M Andrä I Annunziato F. Guidelines for the Use of Flow Cytometry and Cell Sorting in Immunological Studies. Eur J Immunol (2017) 47(10):1584–797. doi: 10.1002/eji.201646632 PMC9165548 29023707

[27] Laskowski TJ Hazen AL Collazo RS Haviland D. Rigor and Reproducibility of Cytometry Practices for Immuno-Oncology: A Multifaceted Challenge. Cytom Part J Int Soc Anal Cytol (2020) 97(2):116–25. doi: 10.1002/cyto.a.23882 31454153

[28] Olsen LR Leipold MD Pedersen CB Maecker HT. The Anatomy of Single Cell Mass Cytometry Data. Cytometry A (2019) 95(2):156–72. doi: 10.1002/cyto.a.23621 30277658

[29] Han G Spitzer MH Bendall SC Fantl WJ Nolan GP. Metal-Isotope-Tagged Monoclonal Antibodies for High-Dimensional Mass Cytometry. Nat Protoc (2018) 13(10):2121–48. doi: 10.1038/s41596-018-0016-7 PMC7075473 30258176

[30] Hartmann FJ Simonds EF Vivanco N Bruce T Borges L Nolan GP. Scalable Conjugation and Characterization of Immunoglobulins With Stable Mass Isotope Reporters for Single-Cell Mass Cytometry Analysis. Methods Mol Biol Clifton NJ (2019) 1989:55–81. doi: 10.1007/978-1-4939-9454-0_5 PMC6687300 31077099

[31] Salesforce. Available at: https://fluidigm.my.salesforce.com/sfc/p/#700000009DAw/a/4u0000019hJc/bT0Mr1sBIRKJDSm14.zvdfrWEJd_QAmX.36RviKDyO8 (Accessed cited 2022 Jan 7).

[32] Han G Spitzer MH Bendall SC Fantl WJ Nolan GP. Metal-Isotope-Tagged Monoclonal Antibodies for High-Dimensional Mass Cytometry. Nat Protoc (2018) 13(10):2121–48. doi: 10.1038/s41596-018-0016-7 PMC7075473 30258176

[33] Zunder ER Finck R Behbehani GK Amir ED Krishnaswamy S Gonzalez VD. Palladium-Based Mass Tag Cell Barcoding With a Doublet-Filtering Scheme and Single-Cell Deconvolution Algorithm. Nat Protoc (2015) 10(2):316–33. doi: 10.1038/nprot.2015.020 PMC4347881 25612231

[34] Mei HE Leipold MD Maecker HT. Platinum-Conjugated Antibodies for Application in Mass Cytometry. Cytometry A (2016) 89(3):292–300. doi: 10.1002/cyto.a.22778 26355391

[35] Han G Chen S-Y Gonzalez VD Zunder ER Fantl WJ Nolan GP. Atomic Mass Tag of Bismuth-209 for Increasing the Immunoassay Multiplexing Capacity of Mass Cytometry. Cytometry A (2017) 91(12):1150–63. doi: 10.1002/cyto.a.23283 PMC5802970 29205767

[36] Leipold MD Newell EW Maecker HT. Multiparameter Phenotyping of Human PBMCs Using Mass Cytometry. Methods Mol Biol Clifton NJ (2015) 1343:81–95. doi: 10.1007/978-1-4939-2963-4_7 PMC4748856 26420710

[37] Qdot Probes Technology Overview - Us (Accessed cited 2021 Oct 31).

[38] Schulz AR Stanislawiak S Baumgart S Grützkau A Mei HE. Silver Nanoparticles for the Detection of Cell Surface Antigens in Mass Cytometry. Cytometry A (2017) 91(1):25–33. doi: 10.1002/cyto.a.22904 27351740

[39] Mitchell AJ Ivask A Ju Y. Quantitative Measurement of Cell-Nanoparticle Interactions Using Mass Cytometry. In: McGuire HM Ashhurst TM, editors. Mass Cytometry: Methods and Protocols. New York, NY: Springer (2019). p. 227–41. doi: 10.1007/978-1-4939-9454-0_15. 31077109

[40] Zhang Y Zabinyakov N Majonis D Bouzekri A Ornatsky O Baranov V. Tantalum Oxide Nanoparticle-Based Mass Tag for Mass Cytometry. Anal Chem (2020) 92(8):5741–9. doi: 10.1021/acs.analchem.9b04970 32239915

[41] Salesforce. Available at: https://fluidigm.my.salesforce.com/sfc/p/#700000009DAw/a/4u0000019hJN/ckomrW7k0trcTk.74DeMn4trwp4hMzDB4d2syo633QA (Accessed cited 2022 Jan 7).

[42] Hartmann FJ Simonds EF Bendall SC. A Universal Live Cell Barcoding-Platform for Multiplexed Human Single Cell Analysis. Sci Rep (2018) 8(1):10770. doi: 10.1038/s41598-018-28791-2 30018331 PMC6050312

[43] McCarthy RL Duncan AD Barton MC. Sample Preparation for Mass Cytometry Analysis. JoVE J Vis Exp (2017) 122):e54394. doi: 10.3791/54394 PMC5565122 28518070

[44] Salesforce. Available at: https://fluidigm.my.salesforce.com/sfc/p/#700000009DAw/a/4u0000019hIZ/saBkuTIq2z0qxYCUmqWxOlbBOYEyNVyNTaMMOxTlU_c (Accessed cited 2022 Jan 7).

[45] Kleinsteuber K Corleis B Rashidi N Nchinda N Lisanti A Cho JL. Standardization and Quality Control for High-Dimensional Mass Cytometry Studies of Human Samples. Cytom Part J Int Soc Anal Cytol (2016) 89(10):903–13. doi: 10.1002/cyto.a.22935 PMC5495108 27575385

[46] Flow Cytometry Controls. Available at: https://www.biolegend.com/en-us/flow-controls?gclid=CjwKCAjwgviIBhBkEiwA10D2j4b6oeL_W1hjQA78FQO-19QijJMgoO0olY9uI_nB4rPecypJToGfKRoCT5AQAvD_BwE#vericellscontrols (Accessed cited 2021 Oct 31).

[47] Bodenmiller B Zunder ER Finck R Chen TJ Savig ES Bruggner RV. Multiplexed Mass Cytometry Profiling of Cellular States Perturbed by Small-Molecule Regulators. Nat Biotechnol (2012) 30(9):858–67. doi: 10.1038/nbt.2317 PMC3627543 22902532

[48] Behbehani GK Thom C Zunder ER Finck R Gaudilliere B Fragiadakis GK. Transient Partial Permeabilization With Saponin Enables Cellular Barcoding Prior to Surface Marker Staining. Cytometry A (2014) 85(12):1011–9. doi: 10.1002/cyto.a.22573 PMC4361015 25274027

[49] Mei HE Leipold MD Schulz AR Chester C Maecker HT. Barcoding of Live Human Peripheral Blood Mononuclear Cells for Multiplexed Mass Cytometry. J Immunol (2015) 194(4):2022–31. doi: 10.4049/jimmunol.1402661 PMC4323739 25609839

[50] Salesforce. Available at: https://fluidigm.my.salesforce.com/sfc/p/#700000009DAw/a/4u0000019jaV/uBxiESdB_ntOGzkDudl9o9kgjSIs4Dad2zFcCyMpb2g (Accessed cited 2022 Jan 7).

[51] Salesforce. Available at: https://fluidigm.my.salesforce.com/sfc/p/#700000009DAw/a/4u0000019ciu/wcEGoi7o1G3CZRiJ_fvF2E_k8VtEDT7am.oALLeTfe4 (Accessed cited 2022 Jan 7).

[52] Muftuoglu M Li L Liang S Mak D Lin AJ Fang J. Extended Live-Cell Barcoding Approach for Multiplexed Mass Cytometry. Sci Rep (2021) 11(1):12388. doi: 10.1038/s41598-021-91816-w 34117319 PMC8196040

[53] McCarthy RL Mak DH Burks JK Barton MC. Rapid Monoisotopic Cisplatin Based Barcoding for Multiplexed Mass Cytometry. Sci Rep (2017) 7(1):3779. doi: 10.1038/s41598-017-03610-2 28630464 PMC5476666

[54] Cell-ID™ Cisplatin, 100 µl. Available at: https://store.fluidigm.com/Cytometry/ConsumablesandReagentsCytometry/MassCytometryReagents/Cell-ID%E2%84%A2%20Cisplatin-%20100%20%C2%B5L?cclcl=en_US (Accessed cited 2022 Jan 7).

[55] Willis LM Park H Watson MWL Majonis D Watson JL Nitz M. Tellurium-Based Mass Cytometry Barcode for Live and Fixed Cells. Cytom Part J Int Soc Anal Cytol (2018) 93(7):685–94. doi: 10.1002/cyto.a.23495 30053343

[56] Catena R Özcan A Zivanovic N Bodenmiller B. Enhanced Multiplexing in Mass Cytometry Using Osmium and Ruthenium Tetroxide Species. Cytometry A (2016) 89(5):491–7. doi: 10.1002/cyto.a.22848 27018769

[57] Gonder S Fernandez Botana I Wierz M Pagano G Gargiulo E Cosma A. Method for the Analysis of the Tumor Microenvironment by Mass Cytometry: Application to Chronic Lymphocytic Leukemia. Front Immunol (2020) 11:2730. doi: 10.3389/fimmu.2020.578176 PMC7606286 33193376

[58] Tung JW Heydari K Tirouvanziam R Sahaf B Parks DR Herzenberg LA. Modern Flow Cytometry: A Practical Approach. Clin Lab Med (2007) 27(3):453–v. doi: 10.1016/j.cll.2007.05.001 PMC1994577 17658402

[59] Oldaker TA Wallace PK Barnett D. Flow Cytometry Quality Requirements for Monitoring of Minimal Disease in Plasma Cell Myeloma. Cytometry B Clin Cytom (2016) 90(1):40–6. doi: 10.1002/cyto.b.21276 PMC5404813 26201282

[60] Drescher H Weiskirchen S Weiskirchen R. Flow Cytometry: A Blessing and a Curse. Biomedicines (2021) 9(11):1613. doi: 10.3390/biomedicines9111613 34829841 PMC8615642

[61] Winkels H Ehinger E Vassallo M Buscher K Dinh HQ Kobiyama K. Atlas of the Immune Cell Repertoire in Mouse Atherosclerosis Defined by Single-Cell RNA-Sequencing and Mass Cytometry. Circ Res (2018) 122(12):1675–88. doi: 10.1161/CIRCRESAHA.117.312513 PMC5993603 29545366

[62] Giesen C Wang HAO Schapiro D Zivanovic N Jacobs A Hattendorf B. Highly Multiplexed Imaging of Tumor Tissues With Subcellular Resolution by Mass Cytometry. Nat Methods (2014) 11(4):417–22. doi: 10.1038/nmeth.2869 24584193

[63] Imaging Mass Cytometry. Available at: https://www.fluidigm.com/products-services/technologies/imaging-mass-cytometry (Accessed cited 2022 Jan 7).

[64] Angelo M Bendall SC Finck R Hale MB Hitzman C Borowsky AD. Multiplexed Ion Beam Imaging of Human Breast Tumors. Nat Med (2014) 20(4):436–42. doi: 10.1038/nm.3488 PMC4110905 24584119

[65] How It Works | MIBI Multiplexed Ion Beam Imaging Technology. Ionpath. Available at: https://www.ionpath.com/mibi-technology/ (Accessed cited 2021 Oct 31).

[66] Protocols for MIBI Multiplexed Tissue Imaging. Ionpath. Available at: https://www.ionpath.com/protocols/ (Accessed cited 2021 Oct 31).

[67] Takahashi C Au-Yeung A Fuh F Ramirez-Montagut T Bolen C Mathews W. Mass Cytometry Panel Optimization Through the Designed Distribution of Signal Interference. Cytometry A (2017) 91(1):39–47. doi: 10.1002/cyto.a.22977 27632576

[68] MACS® Marker Screen, Human, Version 02 | Phenotyping Assays | Kits and Support Reagents | MACS Flow Cytometry | Products | Miltenyi Biotec | USA. Available at: https://www.miltenyibiotec.com/US-en/products/macs-marker-screen-human-version-02.html#gref (Accessed cited 2021 Oct 31).

[69] Amir ED Lee B Badoual P Gordon M Guo XV Merad M. Development of a Comprehensive Antibody Staining Database Using a Standardized Analytics Pipeline. Front Immunol (2019) 10:1315. doi: 10.3389/fimmu.2019.01315 31244854 PMC6579881

[70] Leong ML Newell EW. Multiplexed Peptide-MHC Tetramer Staining With Mass Cytometry. Methods Mol Biol Clifton NJ (2015) 1346:115–31. doi: 10.1007/978-1-4939-2987-0_9 26542719

[71] Leipold MD Ornatsky O Baranov V Whitfield C Nitz M. Development of Mass Cytometry Methods for Bacterial Discrimination. Anal Biochem (2011) 419(1):1–8. doi: 10.1016/j.ab.2011.07.035 21871432

[72] Edgar LJ Vellanki RN Halupa A Hedley D Wouters BG Nitz M. Identification of Hypoxic Cells Using an Organotellurium Tag Compatible With Mass Cytometry. Angew Chem Int Ed Engl (2014) 53(43):11473–7. doi: 10.1002/anie.201405233 25195589

[73] Yang Y-SS Atukorale PU Moynihan KD Bekdemir A Rakhra K Tang L. High-Throughput Quantitation of Inorganic Nanoparticle Biodistribution at the Single-Cell Level Using Mass Cytometry. Nat Commun (2017) 8(1):14069. doi: 10.1038/ncomms14069 28094297 PMC5247578

[74] Frei AP Bava F-A Zunder ER Hsieh EWY Chen S-Y Nolan GP. Highly Multiplexed Simultaneous Detection of RNAs and Proteins in Single Cells. Nat Methods (2016) 13(3):269–75. doi: 10.1038/nmeth.3742 PMC4767631 26808670

[75] Mavropoulos A Allo B He M Park E Majonis D Ornatsky O. Simultaneous Detection of Protein and mRNA in Jurkat and KG-1a Cells by Mass Cytometry. Cytometry A (2017) 91(12):1200–8. doi: 10.1002/cyto.a.23281 29194963

[76] Shaklee J Srivastava K Brown H Arriaga EA Pierre VC van Berlo JH. Development of a Click-Chemistry Reagent Compatible With Mass Cytometry. Sci Rep (2018) 8(1):6657. doi: 10.1038/s41598-018-25000-y 29703991 PMC5923286

[77] Bassan J Willis LM Vellanki RN Nguyen A Edgar LJ Wouters BG. TePhe, a Tellurium-Containing Phenylalanine Mimic, Allows Monitoring of Protein Synthesis *In Vivo* With Mass Cytometry. Proc Natl Acad Sci (2019) 116(17):8155–60. doi: 10.1073/pnas.1821151116 PMC6486722 30971489

[78] Poreba M Groborz KM Rut W Pore M Snipas SJ Vizovisek M. Multiplexed Probing of Proteolytic Enzymes Using Mass Cytometry-Compatible Activity-Based Probes. J Am Chem Soc (2020) 142(39):16704–15. doi: 10.1021/jacs.0c06762 PMC7595764 32870676

[79] Stern AD Rahman AH Birtwistle MR. Cell Size Assays for Mass Cytometry. Cytometry A (2017) 91(1):14–24. doi: 10.1002/cyto.a.23000 27768827 PMC5250533

[80] Good Z Borges L Vivanco Gonzalez N Sahaf B Samusik N Tibshirani R. Proliferation Tracing With Single-Cell Mass Cytometry Optimizes Generation of Stem Cell Memory-Like T Cells. Nat Biotechnol (2019) 37(3):259–66. doi: 10.1038/s41587-019-0033-2 PMC6521980 30742126

[81] Holmberg-Thyden S Grønbæk K Gang AO El Fassi D Hadrup SR. A User’s Guide to Multicolor Flow Cytometry Panels for Comprehensive Immune Profiling. Anal Biochem (2021) 627:114210. doi: 10.1016/j.ab.2021.114210 34033799

[82] Fujita K Maldarelli F Silver J. Bimodal Down-Regulation of CD4 in Cells Expressing Human Immunodeficiency Virus Type 1 Vpu and Env. J Gen Virol (1996) 77(Pt 10):2393–401. doi: 10.1099/0022-1317-77-10-2393 8887470

[83] Bjorkdahl O Barber KA Brett SJ Daly MG Plumpton C Elshourbagy NA. Characterization of CC-Chemokine Receptor 7 Expression on Murine T Cells in Lymphoid Tissues. Immunology (2003) 110(2):170–9. doi: 10.1046/j.1365-2567.2003.01727.x PMC1783047 14511230

[84] Chevrier S Crowell HL Zanotelli VRT Engler S Robinson MD Bodenmiller B. Compensation of Signal Spillover in Suspension and Imaging Mass Cytometry. Cell Syst (2018) 6(5):612–20.e5. doi: 10.1016/j.cels.2018.02.010 29605184 PMC5981006

[85] Schuyler RP Jackson C Garcia-Perez JE Baxter RM Ogolla S Rochford R. Minimizing Batch Effects in Mass Cytometry Data. Front Immunol (2019) 10:2367. doi: 10.3389/fimmu.2019.02367 31681275 PMC6803429

[86] CyTOF FAQ. Available at: https://web.stanford.edu/group/nolan/cytoffaq.html.

[87] Crowell HL Chevrier S Jacobs A Sivapatham S Consortium TP Bodenmiller B. An R-Based Reproducible and User-Friendly Preprocessing Pipeline for CyTOF Data (2020). F1000Research. Available at: https://f1000research.com/articles/9-1263 (Accessed cited 2021 Oct 19). 10.12688/f1000research.26073.1 PMC9411975 36072920

[88] Premessa [Internet]. Parker Institute for Cancer Immunotherapy (2021). Available at: https://github.com/ParkerICI/premessa (Accessed cited 2021 Oct 19).

[89] Burel JG Pomaznoy M Lindestam Arlehamn CS Seumois G Vijayanand P Sette A. The Challenge of Distinguishing Cell–Cell Complexes From Singlet Cells in Non-Imaging Flow Cytometry and Single-Cell Sorting. Cytometry A (2020) 97(11):1127–35. doi: 10.1002/cyto.a.24027 PMC7666012 32400942

[90] Fread KI Strickland WD Nolan GP Zunder ER. An updated debarcoding tool for mass cytometry with cell type-specific and cell sample-specific stringency adjustment. Pac Symp Biocomput Pac Symp Biocomput (2017) 22:588–98. doi: 10.1142/9789813207813_0054 27897009

[91] Preprocessing With CATALYST. Available at: https://bioconductor.org/packages/release/bioc/vignettes/CATALYST/inst/doc/preprocessing.html (Accessed cited 2021 Oct 21).

[92] Bagwell CB Adams EG. Fluorescence Spectral Overlap Compensation for Any Number of Flow Cytometry Parameters. Ann N Y Acad Sci (1993) 677:167–84. doi: 10.1111/j.1749-6632.1993.tb38775.x 8494206

[93] Welcome to CATALYST . catalyst-project.github.io. Available at: https://catalyst-project.github.io/ (Accessed cited 2021 Oct 21).

[94] CytoSpill [Internet]. KChen-Lab. Available at: https://github.com/KChen-lab/CytoSpill (Accessed cited 2021 Oct 21]).

[95] Miao Q Wang F Dou J Iqbal R Muftuoglu M Basar R. Ab Initio Spillover Compensation in Mass Cytometry Data. Cytometry A (2021) 99(9):899–909. doi: 10.1002/cyto.a.24298 33342071 PMC8214634

[96] CyTOForum • View Topic - The New Gaussian Parameters From Helios. Available at: http://cytoforum.stanford.edu/viewtopic.php?f=3&t=709 (Accessed cited 2021 Nov 2).

[97] Bagwell CB Inokuma M Hunsberger B Herbert D Bray C Hill B. Automated Data Cleanup for Mass Cytometry. Cytometry A (2020) 97(2):184–98. doi: 10.1002/cyto.a.23926 31737997

[98] Borg I Groenen PJF. Modern Multidimensional Scaling: Theory and Applications. New York City, United States: Springer Salmon Tower Building (1997). p. 494.

[99] Nowicka M Krieg C Crowell HL Weber LM Hartmann FJ Guglietta S. CyTOF Workflow: Differential Discovery in High-Throughput High-Dimensional Cytometry Datasets(2019) (Accessed cited 2021 Oct 23). 10.12688/f1000research.11622.1 PMC5473464 28663787

[100] Arcsinhtransform: Create the Definition of an Arcsinh Transformation Function … in Flowcore: Flowcore: Basic Structures for Flow Cytometry Data. Available at: https://rdrr.io/bioc/flowCore/man/arcsinhTransform.html (Accessed cited 2021 Oct 27).

[101] Weber LM Robinson MD. Comparison of Clustering Methods for High-Dimensional Single-Cell Flow and Mass Cytometry Data. Cytom Part J Int Soc Anal Cytol (2016) 89(12):1084–96. doi: 10.1002/cyto.a.23030 27992111

[102] Samusik N Good Z Spitzer MH Davis KL Nolan GP. Automated Mapping of Phenotype Space With Single-Cell Data. Nat Methods (2016) 13(6):493–6. doi: 10.1038/nmeth.3863 PMC4896314 27183440

[103] Kimball AK Oko LM Bullock BL Nemenoff RA van Dyk LF Clambey ET. A Beginner’s Guide To Analyzing and Visualizing Mass Cytometry Data. J Immunol Baltim Md 1950 (2018) 200(1):3–22. doi: 10.4049/jimmunol.1701494 PMC5765874 29255085

[104] Levine JH Simonds EF Bendall SC Davis KL Amir ED Tadmor MD. Data-Driven Phenotypic Dissection of AML Reveals Progenitor-Like Cells That Correlate With Prognosis. Cell (2015) 162(1):184–97. doi: 10.1016/j.cell.2015.05.047 PMC4508757 26095251

[105] Van Gassen S Callebaut B Van Helden MJ Lambrecht BN Demeester P Dhaene T. FlowSOM: Using Self-Organizing Maps for Visualization and Interpretation of Cytometry Data. Cytometry A (2015) 87(7):636–45. doi: 10.1002/cyto.a.22625 25573116

[106] Quintelier K Couckuyt A Emmaneel A Aerts J Saeys Y Van Gassen S. Analyzing High-Dimensional Cytometry Data Using FlowSOM. Nat Protoc (2021) 16(8):3775–801. doi: 10.1038/s41596-021-00550-0 34172973

[107] Differential Discovery With CATALYST. Available at: https://bioconductor.org/packages/release/bioc/vignettes/CATALYST/inst/doc/differential.html (Accessed cited 2021 Oct 30).

[108] Magis D Facon B. Deltaplotr : An R Package for Differential Item Functioning Analysis With Angoff’s Delta Plot. J Stat Software (2014) 59:1–19. doi: 10.18637/jss.v059.c01

[109] Salesforce. . Available at: https://fluidigm.my.salesforce.com/sfc/p/#700000009DAw/a/4u0000019krC/EiKTSn7AXdC3eb22YIPeEzdvgDrArze9uzS4XcL8R6s (Accessed cited 2022 Jan 7).

[110] Salesforce. Available at: https://fluidigm.my.salesforce.com/sfc/p/#700000009DAw/a/4u0000019hJh/XL08UnxANI2UDN5B5zAe9X0.vlS1tGQa_P5Qaptya0I (Accessed cited 2022 Jan 7).

[111] BSA Removal Kit. Available at: https://www.thermofisher.com/order/catalog/product/44600.

[112] CyTOForum • View Topic - Stability of Maxpar Conjugated Antibodies. Available at: http://cytoforum.stanford.edu/viewtopic.php?f=7&t=942&hilit=antibody+stability.

[113] Kalina T Lundsten K Engel P. Relevance of Antibody Validation for Flow Cytometry. Cytom Part J Int Soc Anal Cytol (2020) 97(2):126–36. doi: 10.1002/cyto.a.23895 31577065

[114] van Vreden C Niewold P McGuire HM Fazekas de St. Groth B. Titration of Mass Cytometry Reagents. In: McGuire HM Ashhurst TM, editors. Mass Cytometry: Methods and Protocols. New York, NY: Springer (2019). p. 83–92. doi: 10.1007/978-1-4939-9454-0_6. 31077100

[115] Gullaksen S-E Bader L Hellesøy M Sulen A Fagerholt OHE Engen CB. Titrating Complex Mass Cytometry Panels. Cytom Part J Int Soc Anal Cytol (2019) 95(7):792–6. doi: 10.1002/cyto.a.23751 PMC6766997 30964237

[116] Maecker H Trotter J. Selecting Reagents for Multicolor Flow Cytometry With BD™ LSR II and BD FACSCanto™ Systems. Nat Methods (2008) 5(12):an6–7. doi: 10.1038/nmeth.f.229

[117] Nicholas KJ Greenplate AR Flaherty DK Matlock BK Juan JS Smith RM. Multiparameter Analysis of Stimulated Human Peripheral Blood Mononuclear Cells: A Comparison of Mass and Fluorescence Cytometry. Cytom Part J Int Soc Anal Cytol (2016) 89(3):271–80. doi: 10.1002/cyto.a.22799 PMC4808335 26599989

[118] Leipold MD. Another Step on the Path to Mass Cytometry Standardization. Cytometry A (2015) 87(5):380–2. doi: 10.1002/cyto.a.22661 25904393

[119] Cell-ID Intercalator-103Rh—2000 µm. Available at: https://www.fluidigm.com/binaries/content/documents/fluidigm/resources/cell-id-intercalator-103rh-500-um-pi-201103a/cell-id-intercalator-103rh-500-um-pi-201103a/fluidigm:file (Accessed cited 2021 Oct 31).

[120] Spurgeon BEJ Michelson AD Frelinger AL III. Platelet Mass Cytometry: Optimization of Sample, Reagent, and Analysis Parameters. Cytometry A (2021) 99(2):170–9. doi: 10.1002/cyto.a.24300 33399275

[121] Rahman AH Tordesillas L Berin MC. Heparin Reduces Nonspecific Eosinophil Staining Artifacts in Mass Cytometry Experiments. Cytometry A (2016) 89(6):601–7. doi: 10.1002/cyto.a.22826 PMC4919163 27061608

[122] McManus D Novaira HJ Hamers AAJ Pillai AB. Isolation of Lamina Propria Mononuclear Cells From Murine Colon Using Collagenase E. J Vis Exp JoVE (2019) 151:10.3791/59821. doi: 10.3791/59821 PMC7759444 31609324

[123] Leelatian N Doxie DB Greenplate AR Mobley BC Lehman JM Sinnaeve J. Single Cell Analysis of Human Tissues and Solid Tumors With Mass Cytometry. Cytometry B Clin Cytom (2017) 92(1):68–78. doi: 10.1002/cyto.b.21481 27598832 PMC5459378

[124] Korin B Dubovik T Rolls A. Mass Cytometry Analysis of Immune Cells in the Brain. Nat Protoc (2018) 13(2):377–91. doi: 10.1038/nprot.2017.155 29370157

[125] Reichard A Asosingh K. Best Practices for Preparing a Single Cell Suspension From Solid Tissues for Flow Cytometry. Cytom Part J Int Soc Anal Cytol (2019) 95(2):219–26. doi: 10.1002/cyto.a.23690 PMC6375754 30523671

[126] Guldner IH Golomb SM Wang Q Wang E Zhang S. Isolation of Mouse Brain-Infiltrating Leukocytes for Single Cell Profiling of Epitopes and Transcriptomes. STAR Protoc (2021) 2(2):100537. doi: 10.1016/j.xpro.2021.100537 34036283 PMC8138863

[127] David BA Rubino S Moreira TG Freitas-Lopes MA Araújo AM Paul NE. Isolation and High-Dimensional Phenotyping of Gastrointestinal Immune Cells. Immunology (2017) 151(1):56–70. doi: 10.1111/imm.12706 28039862 PMC5382328

[128] Donlin LT Rao DA Wei K Slowikowski K McGeachy MJ Turner JD. Methods for High-Dimensional Analysis of Cells Dissociated From Cryopreserved Synovial Tissue. Arthritis Res Ther (2018) 20(1):139. doi: 10.1186/s13075-018-1631-y 29996944 PMC6042350

[129] Tantalo D Nguyen T Yeang HXA Zhu J Macdonald S Wang M. Using Mass Cytometry to Analyze the Tumor-Infiltrating Lymphocytes in Human Melanoma. Methods Mol Biol Clifton NJ (2021) 2265:543–55. doi: 10.1007/978-1-0716-1205-7_38 33704739

[130] Anandan S Thomsen LCV Gullaksen S-E Abdelaal T Kleinmanns K Skavland J. Phenotypic Characterization by Mass Cytometry of the Microenvironment in Ovarian Cancer and Impact of Tumor Dissociation Methods. Cancers (2021) 13(4):755. doi: 10.3390/cancers13040755 33670410 PMC7918057

[131] Stensland ZC Smith MJ. Enrichment and Detection of Antigen-Binding B Cells for Mass Cytometry. Magnetochem Basel Switz (2021) 7(7):92. doi: 10.3390/magnetochemistry7070092 PMC8294334 34295938

[132] Hassani M Hellebrekers P Chen N van Aalst C Bongers S Hietbrink F. On the Origin of Low-Density Neutrophils. J Leukoc Biol (2020) 107(5):809–18. doi: 10.1002/JLB.5HR0120-459R PMC7318192 32170882

[133] Lemieux J Jobin C Simard C Néron S. A Global Look Into Human T Cell Subsets Before and After Cryopreservation Using Multiparametric Flow Cytometry and Two-Dimensional Visualization Analysis. J Immunol Methods (2016) 434:73–82. doi: 10.1016/j.jim.2016.04.010 27129808

[134] Zhang W Nilles TL Johnson JR Margolick JB. The Effect of Cellular Isolation and Cryopreservation on the Expression of Markers Identifying Subsets of Regulatory T Cells. J Immunol Methods (2016) 431:31–7. doi: 10.1016/j.jim.2016.02.004 PMC4792753 26855370

[135] Weinberg A Song L-Y Wilkening C Sevin A Blais B Louzao R. Optimization and Limitations of Use of Cryopreserved Peripheral Blood Mononuclear Cells for Functional and Phenotypic T-Cell Characterization. Clin Vaccine Immunol CVI (2009) 16(8):1176–86. doi: 10.1128/CVI.00342-08 PMC2725535 19515870

[136] Wang L Hückelhoven A Hong J Jin N Mani J Chen B. Standardization of Cryopreserved Peripheral Blood Mononuclear Cells Through a Resting Process for Clinical Immunomonitoring–Development of an Algorithm. Cytom Part J Int Soc Anal Cytol (2016) 89(3):246–58. doi: 10.1002/cyto.a.22813 26848928

[137] Rosa P Mantovani S Rosboch R Huttner WB. Monensin and Brefeldin A Differentially Affect the Phosphorylation and Sulfation of Secretory Proteins. J Biol Chem (1992) 267(17):12227–32. doi: 10.1016/S0021-9258(19)49828-1 1601888

[138] Schuerwegh AJ Stevens WJ Bridts CH De Clerck LS. Evaluation of Monensin and Brefeldin A for Flow Cytometric Determination of Interleukin-1 Beta, Interleukin-6, and Tumor Necrosis Factor-Alpha in Monocytes. Cytometry (2001) 46(3):172–6. doi: 10.1002/cyto.1102 11449408

[139] Vicetti Miguel RD Maryak SA Cherpes TL Brefeldin A. But Not Monensin, Enables Flow Cytometric Detection of Interleukin-4 Within Peripheral T Cells Responding to Ex Vivo Stimulation With Chlamydia Trachomatis. J Immunol Methods (2012) 384(1–2):191–5. doi: 10.1016/j.jim.2012.07.018 PMC3444442 22850275

[140] Ford T Wenden C Mbekeani A Dally L Cox JH Morin M. Cryopreservation-Related Loss of Antigen-Specific Ifnγ Producing CD4+ T-Cells can Skew Immunogenicity Data in Vaccine Trials: Lessons From a Malaria Vaccine Trial Substudy. Vaccine (2017) 35(15):1898–906. doi: 10.1016/j.vaccine.2017.02.038 PMC5387668 28285985

[141] Fernandez R Maecker H. Cytokine-Stimulated Phosphoflow of Whole Blood Using CyTOF Mass Cytometry. Bio-Protoc (2015) 5(11):e1495–5. doi: 10.21769/BioProtoc.1495 PMC4847742 27135045

[142] Krutzik PO Nolan GP. Intracellular Phospho-Protein Staining Techniques for Flow Cytometry: Monitoring Single Cell Signaling Events. Cytom Part J Int Soc Anal Cytol (2003) 55(2):61–70. doi: 10.1002/cyto.a.10072 14505311

[143] Salesforce. Available at: https://fluidigm.my.salesforce.com/sfc/p/#700000009DAw/a/4u0000019gwX/WXOft4wzySlfbNe.b6WAUQ8sZE2cL7cH_Fl8k93viLk (Accessed cited 2022 Jan 7).

[144] Scalia CR Boi G Bolognesi MM Riva L Manzoni M DeSmedt L. Antigen Masking During Fixation and Embedding, Dissected. J Histochem Cytochem (2017) 65(1):5–20. doi: 10.1369/0022155416673995 27798289 PMC5256198

[145] Coppin E Malergue F Thibult M-L Scifo C Favre C Nunès JA. Flow Cytometric Analysis of Intracellular Phosphoproteins in Human Monocytes. Cytometry B Clin Cytom (2017) 92(3):207–10. doi: 10.1002/cyto.b.21207 25914252

[146] Firaguay G Nunès JA. Analysis of Signaling Events by Dynamic Phosphoflow Cytometry. Sci Signal (2009) 2(86):pl3. doi: 10.1126/scisignal.286pl3 19724061

[147] Andersen MN Al-Karradi SNH Kragstrup TW Hokland M. Elimination of Erroneous Results in Flow Cytometry Caused by Antibody Binding to Fc Receptors on Human Monocytes and Macrophages. Cytom Part J Int Soc Anal Cytol (2016) 89(11):1001–9. doi: 10.1002/cyto.a.22995 27731950

[148] Hamers AAJ Dinh HQ Thomas GD Marcovecchio P Blatchley A Nakao CS. Human Monocyte Heterogeneity as Revealed by High-Dimensional Mass Cytometry. Arterioscler Thromb Vasc Biol (2019) 39(1):25–36. doi: 10.1161/ATVBAHA.118.311022 30580568 PMC6697379

[149] Thomas GD Hamers AAJ Nakao C Marcovecchio P Taylor AM McSkimming C. Human Blood Monocyte Subsets: A New Gating Strategy Defined Using Cell Surface Markers Identified by Mass Cytometry. Arterioscler Thromb Vasc Biol (2017) 37(8):1548–58. doi: 10.1161/ATVBAHA.117.309145 PMC5828170 28596372

[150] Diederichs K. Crystallographic Data and Model Quality. Methods Mol Biol Clifton NJ (2016) 1320:147–73. doi: 10.1007/978-1-4939-2763-0_10 26227042

[151] Capuano C Pighi C Battella S De Federicis D Galandrini R Palmieri G. Harnessing CD16-Mediated NK Cell Functions to Enhance Therapeutic Efficacy of Tumor-Targeting Mabs. Cancers (2021) 13:2500. doi: 10.3390/cancers13102500 34065399 PMC8161310

[152] Lawand M Déchanet-Merville J Dieu-Nosjean M-C. Key Features of Gamma-Delta T-Cell Subsets in Human Diseases and Their Immunotherapeutic Implications. Front Immunol (2017) 8:761. doi: 10.3389/fimmu.2017.00761 28713381 PMC5491929

[153] Wistuba-Hamprecht K Pawelec G Derhovanessian E. OMIP-020: Phenotypic Characterization of Human γδ T-Cells by Multicolor Flow Cytometry. Cytometry A (2014) 85(6):522–4. doi: 10.1002/cyto.a.22470 24756989

[154] Zhu YP Padgett L Dinh HQ Marcovecchio P Wu R Hinz D. Preparation of Whole Bone Marrow for Mass Cytometry Analysis of Neutrophil-Lineage Cells. J Vis Exp JoVE (2019) 148:10.3791/59617. doi: 10.3791/59617 PMC6726111 31282876

[155] de Andres B Hagen M Sandor M Verbeek S Rokhlin O Lynch RG. A Regulatory Role for Fc Gamma Receptors (CD16 and CD32) in Hematopoiesis. Immunol Lett (1999) 68(1):109–13. doi: 10.1016/S0165-2478(99)00038-3 10397164

[156] Schoeberl A Gutmann M Theiner S Schaier M Schweikert A Berger W. Cisplatin Uptake in Macrophage Subtypes at the Single-Cell Level by LA-ICP-TOFMS Imaging. Anal Chem (2021) 93(49):16456–65. doi: 10.1021/acs.analchem.1c03442 PMC8674877 34846133

[157] Salesforce. Available at: https://fluidigm.my.salesforce.com/sfc/p/#700000009DAw/a/4u0000019gwY/kAt2wPkiFPIVRiyUicKj_aVUlQjgfgei0lAKG6I2WWY (Accessed cited 2022 Jan 7).

[158] Lin D Gupta S Maecker HT. Intracellular Cytokine Staining on PBMCs Using CyTOF™ Mass Cytometry. Bio-Protoc (2015) 5(1):e1370. doi: 10.21769/BioProtoc.1370 29104886 PMC5669382

[159] Salesforce. Available at: https://fluidigm.my.salesforce.com/sfc/p/#700000009DAw/a/4u0000019gwS/aIjEtbnLznwN.KbwUA9R1_PqDEgUs8hUhhCFl2vCOGM. (Accessed cited 2022 Jan 7)

[160] Simoni Y Fehlings M Newell EW. Multiplex MHC Class I Tetramer Combined With Intranuclear Staining by Mass Cytometry, in: Mass Cytometry: Methods and Protocols (2019). New York, NY: Springer. doi: 10.1007/978-1-4939-9454-0_11 (Accessed cited 2021 Nov 2). Methods in Molecular Biology. 31077105

[161] Salesforce. Available at: https://fluidigm.my.salesforce.com/sfc/p/#700000009DAw/a/4u0000019gvy/k1.CbKFY8JN7swG2ZVHybPpEZldzMpDAZnJYQvxyrrc (Accessed cited 2022 Jan 7).

[162] Schulz AR Baumgart S Schulze J Urbicht M Grützkau A Mei HE. Stabilizing Antibody Cocktails for Mass Cytometry. Cytometry A (2019) 95(8):910–6. doi: 10.1002/cyto.a.23781 31058420

[163] Phospho-Protein Staining Tips. Available at: https://www.fluidigm.com/binaries/content/documents/fluidigm/resources/phospho-protein-staining-tips/phospho-protein-staining-tips/fluidigm%3Afile (Accessed cited 2021 Nov 2).

[164] Cosma A. The Nightmare of a Single Cell: Being a Doublet. Cytom Part J Int Soc Anal Cytol (2020) 97(8):768–71. doi: 10.1002/cyto.a.23929 31743590

[165] Sumatoh HR Teng KWW Cheng Y Newell EW. Optimization of Mass Cytometry Sample Cryopreservation After Staining. Cytom Part J Int Soc Anal Cytol (2017) 91(1):48–61. doi: 10.1002/cyto.a.23014 27798817

[166] Leipold MD Maecker HT. Mass Cytometry: Protocol for Daily Tuning and Running Cell Samples on a CyTOF Mass Cytometer. JoVE J Vis Exp (2012) 69):e4398. doi: 10.3791/4398 PMC3499083 23149654

[167] CyTOF 2 User Manual. Available at: https://www.fluidigm.com/binaries/content/documents/fluidigm/resources/cytof-2-mass-cytometer/cytof-2-mass-cytometer/fluidigm%3Afile (Accessed cited 2021 Oct 31).

[168] CyTOForum • View Topic - Long CyTOF Runs in Helios. Available at: http://cytoforum.stanford.edu/viewtopic.php?f=1&t=1700 (Accessed cited 2022 Jan 7).

[169] CyTOForum • View Topic - Sudden Signal Intensity Drop During Helios Runs. Available at: http://cytoforum.stanford.edu/viewtopic.php?f=4&t=946 (Accessed cited 2022 Jan 7).

[170] Lee BH Kelly G Bradford S Davila M Guo XV Amir E-AD. A Modified Injector and Sample Acquisition Protocol Can Improve Data Quality and Reduce Inter-Instrument Variability of the Helios Mass Cytometer. Cytometry A (2019) 95(9):1019–30. doi: 10.1002/cyto.a.23866 PMC6750971 31364278

[171] Finck R Simonds EF Jager A Krishnaswamy S Sachs K Fantl W. Normalization of Mass Cytometry Data With Bead Standards. Cytom Part J Int Soc Anal Cytol (2013) 83(5):483–94. doi: 10.1002/cyto.a.22271 PMC3688049 23512433

[172] EQ Four Element Calibration Beads—100 Ml. Available at: https://store.fluidigm.com/Cytometry/ConsumablesandReagentsCytometry/MaxparBuffersAndSolutions/EQ%20Four%20Element%20Calibration%20Beads%E2%80%94100%20mL?cclcl=en_US (Accessed cited 2022 Jan 7).

[173] Tricot S Meyrand M Sammicheli C Elhmouzi-Younes J Corneau A Bertholet S. Evaluating the Efficiency of Isotope Transmission for Improved Panel Design and a Comparison of the Detection Sensitivities of Mass Cytometer Instruments. Cytom Part J Int Soc Anal Cytol (2015) 87(4):357–68. doi: 10.1002/cyto.a.22648 25704858

[174] Leipold MD Obermoser G Fenwick C Kleinstuber K Rashidi N McNevin JP. Comparison of CyTOF Assays Across Sites: Results of a Six-Center Pilot Study. J Immunol Methods (2018) 453:37–43. doi: 10.1016/j.jim.2017.11.008 29174717 PMC5805584

[175] Liu J Jarzabek J Majonis D Watson J Baranov V Winnik MA. Metal-Encoded Polystyrene Microbeads as a Mass Cytometry Calibration/Normalization Standard Covering Channels From Yttrium (89 Amu) to Bismuth (209 Amu). Anal Chem (2020) 92(1):999–1006. doi: 10.1021/acs.analchem.9b03935 31815445

[176] Data Scientist’s Primer to Analysis of Mass Cytometry Data. Available at: https://biosurf.org/cytof_data_scientist.html (Accessed cited 2021 Oct 19).

[177] Random Function - RDocumentation. Available at: https://www.rdocumentation.org/packages/base/versions/3.6.2/topics/Random (Accessed cited 2021 Oct 30).

[178] Horowitz A Strauss-Albee DM Leipold M Kubo J Nemat-Gorgani N Dogan OC. Genetic and Environmental Determinants of Human NK Cell Diversity Revealed by Mass Cytometry. Sci Transl Med (2013) 5(208):208ra145. doi: 10.1126/scitranslmed.3006702 PMC3918221 24154599

[179] Hsieh W-C Lai E-Y Liu Y-T Wang Y-F Tzeng Y-S Cui L. NK Cell Receptor and Ligand Composition Influences the Clearance of SARS-CoV-2. J Clin Invest (2021) 131(21):e146408. doi: 10.1172/JCI146408 34720095 PMC8553551

[180] Toghi Eshghi S Au-Yeung A Takahashi C Bolen CR Nyachienga MN Lear SP. Quantitative Comparison of Conventional and T-SNE-Guided Gating Analyses. Front Immunol (2019) 10:1194. doi: 10.3389/fimmu.2019.01194 31231371 PMC6560168

[181] Becht E Dutertre C-A Kwok IWH Ng LG Ginhoux F Newell EW. Evaluation of UMAP as an Alternative to T-SNE for Single-Cell Data (2018). Available at: https://www.biorxiv.org/content/10.1101/298430v1 (Accessed cited 2021 Oct 30).

[182] Kraemer G Reichstein M Mahecha MD. Dimred and Coranking - Unifying Dimensionality Reduction in R. R J (2018) 10(1):342–58. doi: 10.32614/RJ-2018-039

[183] Groth D Hartmann S Klie S Selbig J. Principal Components Analysis. Methods Mol Biol Clifton NJ (2013) 930:527–47. doi: 10.1007/978-1-62703-059-5_22 23086856

[184] Heiser CN Lau KS. A Quantitative Framework for Evaluating Single-Cell Data Structure Preservation by Dimensionality Reduction Techniques. Cell Rep (2020) 31(5):107576. doi: 10.1016/j.celrep.2020.107576 32375029 PMC7305633

[185] CyTOF Workflow: Differential Discovery in High-Throughput High-Dimensional Cytometry Datasets. Available at: https://www.bioconductor.org/packages/release/workflows/vignettes/cytofWorkflow/inst/doc/cytofWorkflow.html (Accessed cited 2021 Oct 30). 10.12688/f1000research.11622.1 PMC5473464 28663787

[186] Haghverdi L Buettner F Theis FJ. Diffusion Maps for High-Dimensional Single-Cell Analysis of Differentiation Data. Bioinforma Oxf Engl (2015) 31(18):2989–98. doi: 10.1093/bioinformatics/btv325 26002886

[187] Bendall SC Davis KL Amir ED Tadmor MD Simonds EF Chen TJ. Single-Cell Trajectory Detection Uncovers Progression and Regulatory Coordination in Human B Cell Development. Cell (2014) 157(3):714–25. doi: 10.1016/j.cell.2014.04.005 PMC4045247 24766814

[188] Setty M Tadmor MD Reich-Zeliger S Angel O Salame TM Kathail P. Wishbone Identifies Bifurcating Developmental Trajectories From Single-Cell Data. Nat Biotechnol (2016) 34(6):637–45. doi: 10.1038/nbt.3569 PMC4900897 27136076

[189] Qiu X Mao Q Tang Y Wang L Chawla R Pliner HA. Reversed Graph Embedding Resolves Complex Single-Cell Trajectories. Nat Methods (2017) 14(10):979–82. doi: 10.1038/nmeth.4402 PMC5764547 28825705

[190] Home | FlowJo, LLC. Available at: https://www.flowjo.com/ (Accessed cited 2021 Oct 30).

[191] FCS Express Flow Cytometry Software. De Novo Software. Available at: https://denovosoftware.com/ (Accessed cited 2021 Oct 30).

[192] Chen TJ Kotecha N. Cytobank: Providing an Analytics Platform for Community Cytometry Data Analysis and Collaboration. Curr Top Microbiol Immunol (2014) 377:127–57. doi: 10.1007/82_2014_364 24590675

[193] Cytofkit: An Integrated Mass Cytometry Data Analysis Pipeline (2021). Jinmiao Chen’s Lab. Available at: https://github.com/JinmiaoChenLab/cytofkit (Accessed cited 2021 Oct 30). 10.1371/journal.pcbi.1005112 PMC5035035 27662185

[194] The Astrolabe Cytometry Platform, Solving Single-Cell Analysis . Astrolabe Diagnostics. Available at: https://astrolabediagnostics.com (Accessed cited 2021 Oct 30).

[195] Lee H-C Kosoy R Becker CE Dudley JT Kidd BA. Automated Cell Type Discovery and Classification Through Knowledge Transfer. Bioinformatics (2017) 33(11):1689–95. doi: 10.1093/bioinformatics/btx054 PMC5447237 28158442

[196] Helios User Manual. Available at: https://www.fluidigm.com/binaries/content/documents/fluidigm/resources/helios-user-guide0400250/helios-user-guide0400250/fluidigm%3Afile (Accessed cited 2021 Oct 31).

[197] Polikowsky HG Drake KA. Supervised Machine Learning With CITRUS for Single Cell Biomarker Discovery. Methods Mol Biol Clifton NJ (2019) 1989:309–32. doi: 10.1007/978-1-4939-9454-0_20 PMC7458409 31077114

[198] Bruggner RV Bodenmiller B Dill DL Tibshirani RJ Nolan GP. Automated Identification of Stratifying Signatures in Cellular Subpopulations. Proc Natl Acad Sci (2014) 111(26):E2770–7. doi: 10.1073/pnas.1408792111 PMC4084463 24979804

[199] Traeger JC. Photoionization and Photodissociation Methods in Mass Spectrometry, in: Encyclopedia of Spectroscopy and Spectrometry (Third Edition) (2017). Oxford: Academic Press. Available at: https://www.sciencedirect.com/science/article/pii/B9780128032244002557 (Accessed cited 2021 Nov 2).

[200] Mellon FA. MASS SPECTROMETRY | Principles and Instrumentation, in: Encyclopedia of Food Sciences and Nutrition (Second Edition) (2003). Oxford: Academic Press. Available at: https://www.sciencedirect.com/science/article/pii/B012227055X00746X (Accessed cited 2021 Nov 2).

[201] Abdelaal T Höllt T van Unen V Lelieveldt BPF Koning F Reinders MJT. CyTOFmerge: Integrating Mass Cytometry Data Across Multiple Panels. Bioinformatics (2019) 35(20):4063–71. doi: 10.1093/bioinformatics/btz180 PMC6792069 30874801

[202] Spitzer MH Gherardini PF Fragiadakis GK Bhattacharya N Yuan RT Hotson AN. An Interactive Reference Framework for Modeling a Dynamic Immune System. Science (2015) 349(6244):1259425. doi: 10.1126/science.1259425 26160952 PMC4537647

[203] Van den Berge K Roux de Bézieux H Street K Saelens W Cannoodt R Saeys Y. Trajectory-Based Differential Expression Analysis for Single-Cell Sequencing Data. Nat Commun (2020) 11(1):1201. doi: 10.1038/s41467-020-14766-3 32139671 PMC7058077

[204] Abdelaal T van Unen V Höllt T Koning F Reinders MJT Mahfouz A. Predicting Cell Populations in Single Cell Mass Cytometry Data. Cytometry A (2019) 95(7):769–81. doi: 10.1002/cyto.a.23738 PMC6767556 30861637

[205] Ijsselsteijn ME Somarakis A Lelieveldt BPF Höllt T de Miranda NFCC. Semi-Automated Background Removal Limits Data Loss and Normalizes Imaging Mass Cytometry Data. Cytom Part J Int Soc Anal Cytol (2021) 99(12):1187–97. doi: 10.1002/cyto.a.24480 PMC9542015 34196108

